# Selective Fluorescent Sensing for Iron in Aqueous Solution by A Novel Functionalized Pillar[5]arene

**DOI:** 10.1002/open.202300109

**Published:** 2023-10-06

**Authors:** Yahan Zhang, Longming Chen, Xinbei Du, Xiang Yu, Han Zhang, Zhao Meng, Zhibing Zheng, Junyi Chen, Qingbin Meng

**Affiliations:** ^1^ State Key Laboratory of Toxicology and Medical Countermeasures Beijing Institute of Pharmacology and Toxicology Beijing 100850 P. R. China; ^2^ Key Laboratory of Inorganic-Organic Hybrid Functional Material Chemistry Ministry of Education Tianjin Key Laboratory of Structure and Performance for Functional Molecules College of Chemistry Tianjin Normal University Tianjin 300387 P. R. China

**Keywords:** fluorescent sensor, iron sensing, pillar[n]arene, supramolecular chemistry, ultrasensitive

## Abstract

Iron ion is one of the most physiologically important elements in metabolic processes, indispensable for all living systems. Since its excess can lead to severe diseases, new approaches for its monitoring in water samples are urgently needed to meet requirements. Here, we firstly report a novel and universal route for the synthesis of a series of pillar[n]arene derivates containing one benzoquinone unit by photocatalysis. With this in hand, an anthracene – appended water – soluble pillar[5]arene (**H**) with excellent fluorescence sensing potency was prepared. **H** enabled the ultrasensitive detection of iron ions in aqueous solution with limits of detection of 10^−8^ M. Over a wide range of metal ions, **H** exhibited specific selectivity toward Fe^3+^. More importantly, **H** could still properly operate in a simulated sewage sample, coexisting with multiple interference ions.

## Graphical abstract

An anthracene‐appended water‐soluble pillar[5]arene was prepared to construct a supramolecular fluorescent sensor system to accurately and selectively detect Fe^3+^ in water.

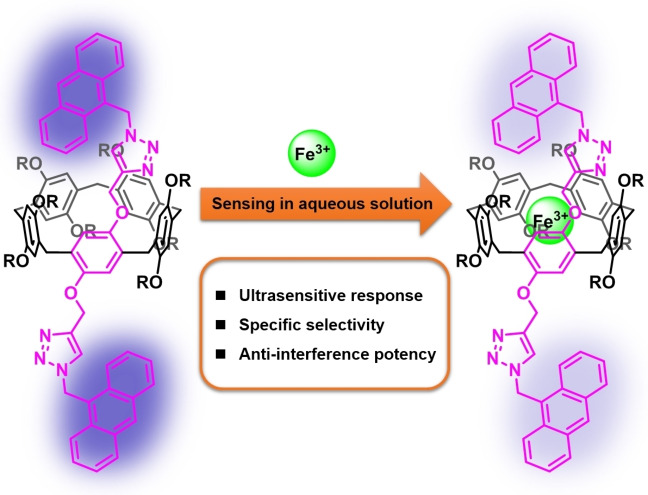

## Introduction

Iron plays essential roles with respect to many biochemical processes including oxygen transport, energy production and enzyme catalysis ascribed to facile redox property and high affinity for oxygen.[^1^, ^2^, ^3^] Despite this central role in biology, excess iron will cause severe cell oxidative damage through aberrant production of highly reactive oxygen species, inducing the occurrence of many diseases, such as cancer and some neurodegenerative diseases.[^4^, ^5^, ^6^] Human beings are unable to excrete iron actively, and its uptake from diets should be severely controlled daily.[^7^, ^8^, ^9^] Among these, the World Health Organization (WHO) has set maximum allowed levels of iron in drinking water to be 0.3 mg L^−1^ (5.4 μM).^[10]^ Some analytical techniques including inductively coupled plasma mass spectrometry and atomic absorption spectroscopy have been employed currently for monitoring its content.[^11^, ^12^, ^13^] While the requirements of sophisticated devices, professional staffs and exorbitant management fees limit their popularization, especially in some low‐income countries.[^14^, ^15^] Thus development of economical, convenient, sensitive approach is urgent needed to meet extensive market demand.

**Scheme 1:**
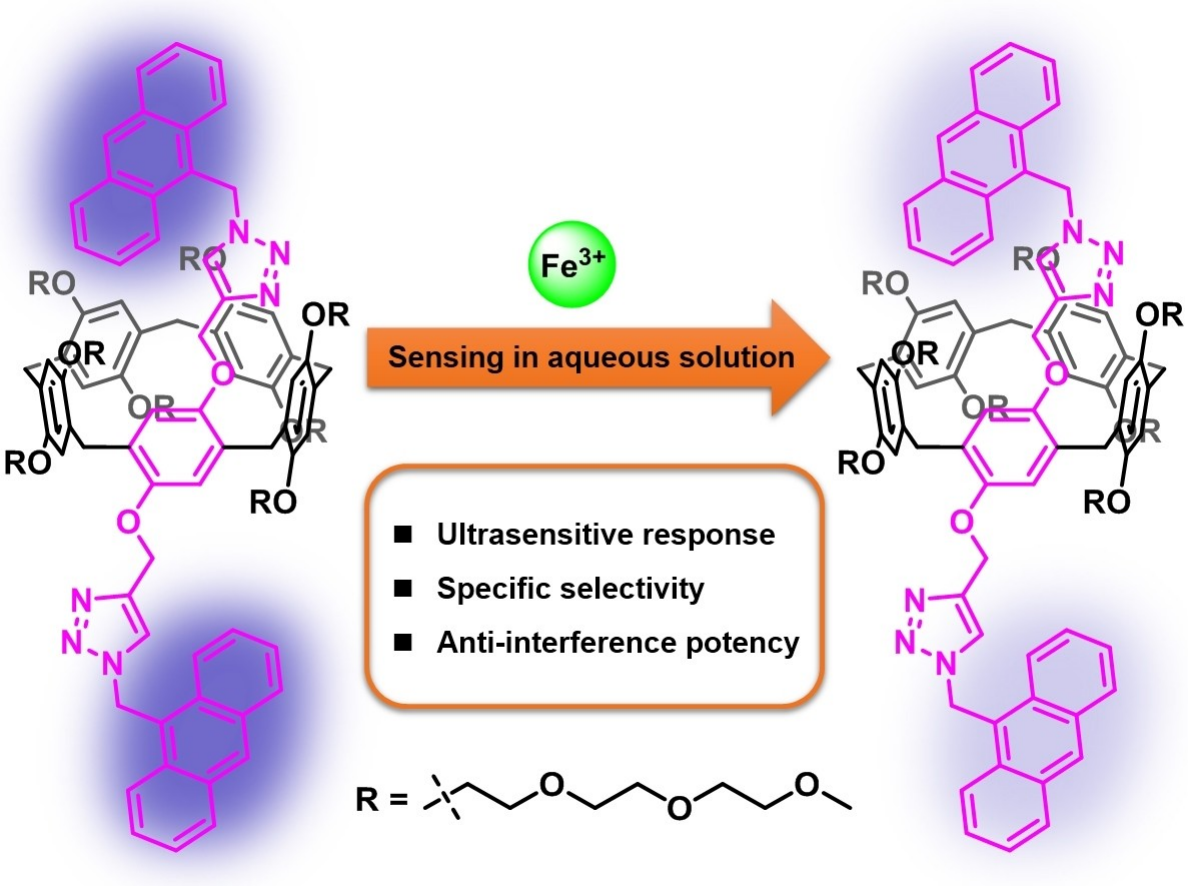
Chemical structure of an anthracene‐appended water‐soluble pillar[5]arene (**H**) and schematic illustration of **H** as supramolecular fluorescent sensor system to accurately and selectively detect Fe^3+^ in aqueous solution.

Supramolecular fluorescent sensor systems based on synthetic macrocycles have been employed as an alternative in trace detection.[^16^, ^17^, ^18^, ^19^, ^20^, ^21^, ^22^] Pillar[n]arenes as an important class of macrocyclic hosts in supramolecular chemistry possess excellent pre‐organized prism structures and host‐guest recognition properties.[^23^, ^24^, ^25^, ^26^] Compared with other macrocycles such as crown ethers, cucurbit[n]uril and calix[n]arenes, pillararenes have advantages in convenient and versatile functionalization similar to cyclodextrins.^[27]^ Recently, a great deal of attention was devoted to design and synthesis partially functionalized pillararenes to build new structures and generate novel applications.[^28^, ^29^, ^30^, ^31^, ^32^, ^33^] For example, a difunctionalized pillar[5]arenes containing two 4,4’‐(1,4‐phenylenedi‐2,1‐ethenediyl) bis‐pyridine (DPB) units was successfully fabricated by Xiao and coworkers to selectively detect Fe^3+^/Ag^+^ in water/DMSO.^[34]^ While these studies mostly focused on pillararenes with linear alkyl chains, partially functionalized materials based on water‐soluble pillararenes are very scarce. Herein, pillar[5]arenes bearing triethylene oxide group (TEP5) was employed as a macrocyclic scaffold benefiting from their facile modification and water solubility. Under ultraviolet light, one of monomer of TEP5 converted to benzoquinone unit and then introduction of anthracene moieties via Huisgen‐type click reaction afforded fluorescence sensor (**H**, Scheme 1). Synthetic sensor **H** with multi‐ion recognition sites was capable to efficiently monitor iron in low μM range with a detection limit of 6.4×10^−8^ M in aqueous solution. More importantly, coexisting with other metal ions had no influence on the sensitiveness of **H**, which endowed its great potential in practical applications.

## Results and Discussion

Notably, there are multiple ways to synthesise per‐functionalized pillar[n]arenes, but selective partial functionalization is still a tough challenge.[^35^, ^36^, ^37^]

**Figure 1:**
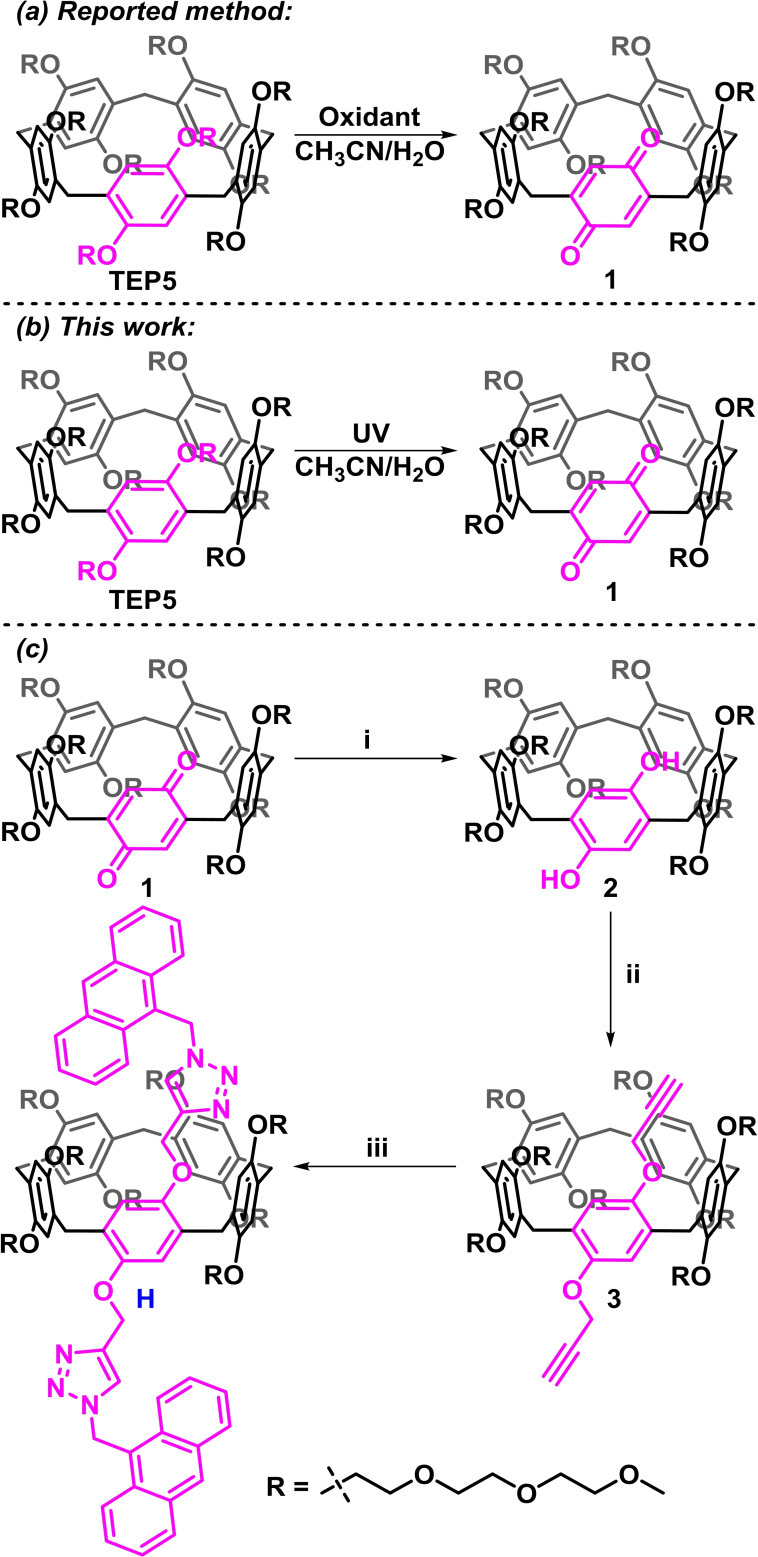
Synthesis of water‐soluble tri(ethylene oxide)‐substituted pillar[5]arene containing one benzoquinone unit (Qui‐TEP5) using (a) hypervalent‐iodine oxidation in the previous work and (b) photocatalytic in this work. (c) Synthetic route to di‐anthracene‐modified pillararene (**H**). Reagents and conditions: i NaBH_4_, MeOH/H_2_O. ii 3‐bromopropyne, K_2_CO_3_, acetone. iii 9‐azidomethylanthracene, Cu(MeCN)_4_PF_6_, triethylamine, DCM.

**Figure 2:**
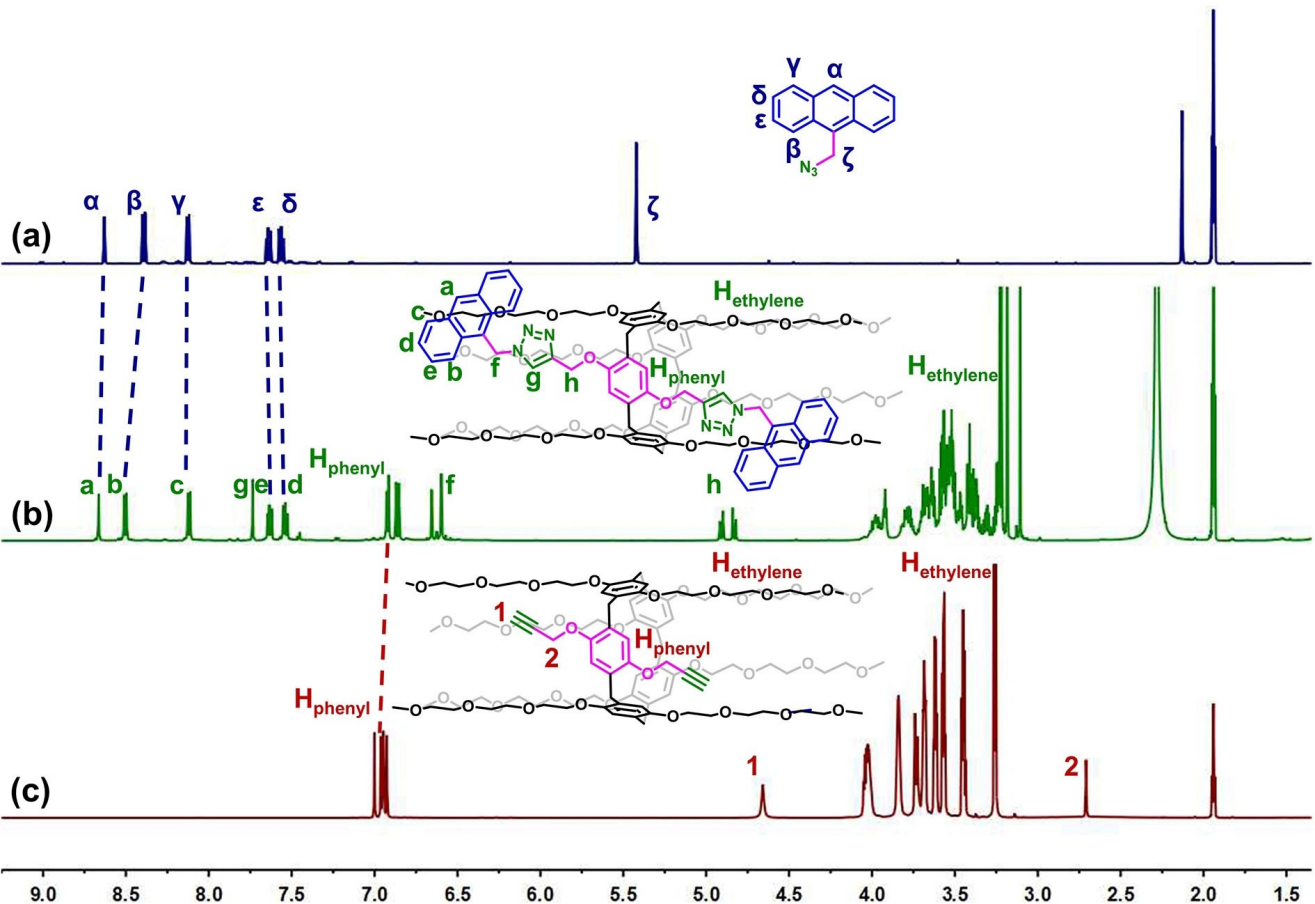
^1^H NMR spectra (600 MHz, CD_3_CN) of (a) 9‐ azidomethylanthracene (b) **H** and (c) compound **3**.

In the previous work, tri(ethylene oxide)‐substituted pillar[5]arene containing one benzoquinone unit (Qui‐TEP5) was synthesized by hypervalent iodine oxidation (Figure 1a), which could be further reacted to introduce functional moieties.^[38]^ The possibly that photocatalysis as an eco‐friendly method could provide an alternative to conventional synthetic pathways has attracted growing interest.^[39]^ Herein, we firstly tried ultraviolet light as an oxidant to successfully obtain Qui‐TEP5 (Figure 1b). Interestingly, we discovered that Qui‐TEP5 could be converted under the absorption wavelength range of pillar[5]arenes bearing a triethylene oxide group, indicating that the reaction was wavelength‐independent (Figure S27). Furthermore, it is noteworthy that this protocol was also compatible with pillar[5]arene bearing oligo‐ethylene oxide chains of varying length and pillar[6]arene derivates. Details of synthetic procedures and compound characterization are shown in the Supporting Information (Scheme S1, Figure S1–18). The synthetic fluorescent sensor was then prepared through a three‐step reaction (Figure 1c). Upon reduction of the benzoquinone moiety by NaBH_4_, compound **2** was obtained. Subsequently, compound **3** was prepared from compound **2** using an excess of 3‐bromopropyne in presence of K_2_CO_3_. Finally, **H** was synthesized by copper(I)‐catalysed Husigen alkyne‐azide 1,3‐dipolar cycloaddition (CuAAC ‘click’ reaction) between compound **3** and 9‐azidomethylanthracene. ^1^H NMR spectroscopic studies were conducted to investigate the functionalization of the pillararenes. As shown in Figure 2, the protons of H_ζ_ of 9‐azidomethylanthracene underwent a significant downfield shift and the protons of H_1_ of compound **3** disappeared and changed to H_g_. These results indicate that a di‐functionalized pillararene was produced by the reaction of compound **3** with 9‐azidomethylanthracene. Details of the synthetic procedures and compound characterization are shown in the Supporting Information (Figure S19–26).

The interaction between **H** and Fe^3+^ was firstly studied by ^1^H NMR spectroscopic titration (Figure S28). Upon addition of Fe^3+^, the peaks for protons H_ethylene_ were shifted to lower field. An up‐field chemical shift was observed for other protons (H_a_, H_b_, H_f_), which was similar to the changes of chemical shift reported in the literature.[^40^, ^41^] These results support the interaction between **H** and Fe^3+^. A fluorescent chemosensor usually involves two components. One is a signalling fluorophore, the other is a guest receptor that possesses a recognition capability.^[14]^

Subsequently, in order to investigate whether pillararenes unfunctionalized with anthracene groups could be a guest receptor, the interaction between TEP5 and Fe^3+^ was verified by ^1^H NMR spectroscopy. As shown in Figure S29, the protons of phenyl group exhibited up‐field chemical shifts, which was in accordance with the results of ^1^H NMR titration, demonstrating pillararenes unfunctionalized with anthracene groups played an important role in the recognition of Fe^3+^.

**Figure 3:**
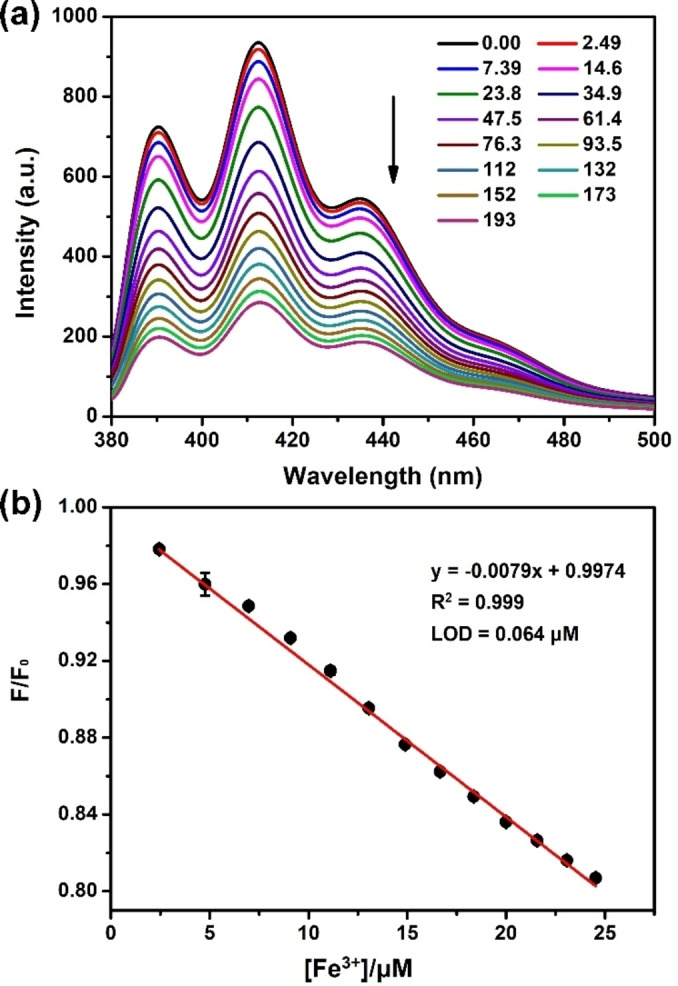
(a) Fluorescence spectra of **H** (20 μM) in the presence of various concentration of Fe^3+^ in water (*λ*_ex_=365 nm). (b) The set‐up calibration line of the fluorescence intensity for quantitatively determining the Fe^3+^ concentration in water.

To evaluate whether synthetic fluorescent sensor **H** could sensitively detect Fe^3+^ in pure water, UV‐visible and fluorescence spectroscopy were employed. Upon addition of 10 equiv. of Fe^3+^, an obvious UV intensity increase was observed in the absorbance band of **H** at 250 nm, 320 nm and 390 nm, indicating strong noncovalent interactions between them (Figure S30). As shown in Figure 3a, **H** displayed a characteristic fluorescence emission band in the 380–500 nm range, which was ascribed to anthracene units. Fe^3+^, a d‐block metal ion can open excited state deexcitation pathways via photo‐induced electronic transfer (PET). Upon addition of Fe^3+^, the fluorescence of **H** was quenched without any shift of the emission maximum, revealing a PET quenching mechanism.[^42^, ^43^] The association constant extracted from the fluorescence titration, was fitted as (3.78±0.20)×10^3^ M^−1^. Additionally, with the mixture of 10 equiv. of Fe^3+^, *I*_free_/*I*_bound_ at 413 nm was calculated as a factor of 3.28 (Figure S31), which was ideal for the projected Fe^3+^ detection in water. Furthermore, a linear decrease (R^2^=0.999) in the fluorescence of **H** (20 μM) was observed upon the gradual increase in Fe^3+^concentrations (Figure 3b). The limits of detection (LODs) of **H** for Fe^3+^ was determined to be 64 ppm based on the 3δ/S method, which was well below the standard concentration of iron set by WHO. Taken together, **H** as a synthetic fluorescent sensor has an ultrasensitive response property toward Fe^3+^.

**Figure 4:**
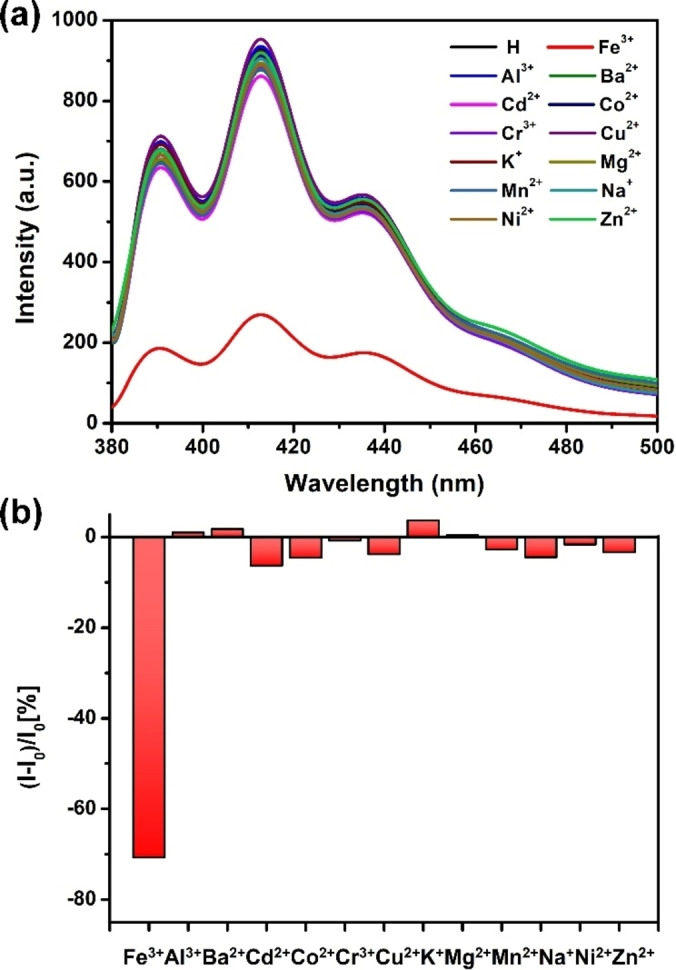
(a) Fluorescence emission spectra for a 1 : 10 mixture of **H** (20 μM) and different metal ions: Fe^3+^, Al^3+^, Ba^2+^, Cd^2+^, Co^2+^, Cr^3+^, Cu^2+^, K^+^, Mg^2+^, Mn^2+^, Na^+^, Ni^2+^, Zn^2+^ as their chloride salts in water (*λ*_ex_=365 nm). (b) Histogram representing the fluorescence response of **H** after the addition of 10 equiv. of various ions in water.

In addition to sensitively respond to Fe^3+^, the ability to selectively detect Fe^3+^ is another requirement for supramolecular fluorescent sensor systems. Next, the selectivity of this synthetic fluorescent sensor toward Fe^3+^ was verified. A 20 μM of **H** solution was prepared and 10 equiv. of metal ions including Fe^3+^, Al^3+^, Ba^2+^, Cd^2+^, Co^2+^, Cr^3+^, Cu^2+^, K^+^, Mg^2+^, Mn^2+^, Na^+^, Ni^2+^, Zn^2+^ were added, respectively. The change of fluorescence intensity by exciting the corresponding solutions at 365 nm is shown in Figure 4a and only Fe^3+^ induced significant fluorescence quenching up to 70 % (Figure 4b). In contrast, none of the other metal ions could induce obvious fluorescence fluctuations of **H**. Competitive experiments were also performed to assess the anti‐interference property of **H** under the condition of coexisting with other metal ions. The fluorescence emission spectra of **H** (20 μM) with 10 equiv. of various other ions (Al^3+^, Ba^2+^, Cd^2+^, Co^2+^, Cr^3+^, Cu^2+^, K^+^, Mg^2+^, Mn^2+^, Na^+^, Ni^2+^, Zn^2+^) were recorded respectively in the absence and presence of 10 equiv. of Fe^3+^.

**Figure 5:**
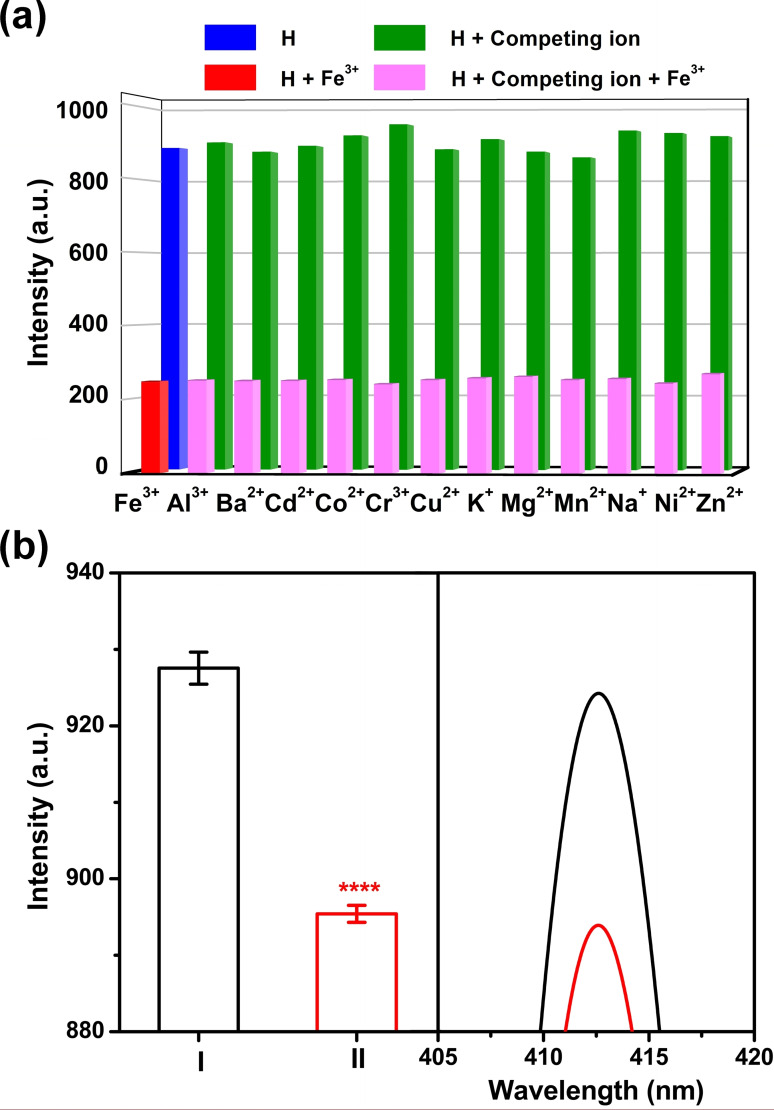
(a) Fluorescence intensity changes of **H** (20 μM) to Fe^3+^ (10 equiv.) in the presence of 10 equiv. of various ions in water (*λ*_em_=413 nm). (b) Left: Fluorescence intensity of I (**H**) and II (the mixture of **H** with 5 μM Fe^3+^) (*λ*_em_=413 nm). Right: Fluorescence emission spectra (*λ*_ex_=365 nm) adding Fe^3+^ (5 μM) into **H** (20 μM) in water. *P‐*values are determined using t‐test. ****P≤0.0001.

As shown in Figure 5a, coexisting with competing ions, Fe^3+^ still effectively quenched the fluorescence of **H**, with a *I*_free_/*I*_bound_ value which was similar to that of **H** alone with Fe^3+^. These results proved that the investigated competing metal ions had little influence on the fluorescence intensity of **H** with Fe^3+^, indicating that **H** exhibited specific fluorescence selectivity toward Fe^3+^.The anti‐interference ability should be attributed to the strong host‐guest interaction between **H** and Fe^3+^. In addition, **H** is decorated with two fluorophores and triazole groups, which provided more chelating sites for ion recognition and contributed to the selectivity to iron(III) cations. Subsequently, we investigated the practical application of **H** for accurate detection of iron concentration in complex environments. Iron(III) cations (5 μM) in aqueous water were prepared to serve as a standard sample for the following test. For comparison, the iron content was determined by a commercial Iron Assay Kit (Adsbio, Jiangsu, China) according to protocols. The appropriate calibration curve was firstly derived (Figure S32) and concentration of Fe^3+^ was calculated to be 2.82±0.83 μmol/L. Following this, synthetic fluorescent sensor **H** was utilized to measure the same sample. As shown in Figure 5b, a significant fluorescence decrease was seen and iron concentration was determined by above established calibration curve to be 4.34±0.16 μmol/L, which were consistent with data of test kit. Furthermore, one equiv. of Zn^2+^, Mg^2+^, Ba^2+^, Al^3+^, Ni^2+^, Co^2+^, Cu^2+^, Cd^2+^, Cr^3+^, Mn^2+^, K^+^ and Na^+^ were added to simulate a sewage sample. In presence of multiple metal ions, **H** was still caught by Fe^3+^ with obvious fluorescence quenching (Figure S33). The measurement results of test kit and fluorescence assay were 3.67±0.27 μmol/L and 6.52±0.19 μmol/L, respectively. Taken together, these favourable findings suggested that a fluorescence assay built by **H** could accurately and selectively detect Fe^3+^ concentration down to low μM range even if coexisting with multiple metal ions, providing new way to conveniently monitor water quality in practical application.

## Conclusions

In summary, a novel anthracene‐appended water‐soluble pillar[5]arene was successfully synthesized. **H** exhibited ultrasensitive potency to detect Fe^3+^ ions in aqueous solution. The LODs value was determined to be 64 ppm by the 3δ/S method, which was lower than the standard level in drinking water set by WHO. Anti‐interference experiments proved that **H** had specific fluorescence selectivity to Fe^3+^ and other metal ions including Zn^2+^, Mg^2+^, Ba^2+^, Al^3+^, Ni^2+^, Co^2+^, Cu^2+^, Cd^2+^, Cr^3+^, Mn^2+^, K^+^ and Na^+^ had nearly no influence on sensing behaviour. More importantly, **H** could accurately detect Fe^3+^ content in a simulated sewage sample and its efficacy was equivalent with a commercial test kit. Overall, these results suggest that this synthetic fluorescent sensor has a potential impact in practically monitoring water sample of Fe^3+^. Studies directed to exploring its possibility of applications in diagnostics and imaging are ongoing in our lab.

## Supporting Information Summary

The authors have cited additional references within the Supporting Information [44–46]. The Supporting Information contains the experimental details for synthesis and fully analytical characterization of the compounds such as NMR and HRMS spectra. Additional UV‐visible spectra and fluorescence emission spectra are also included as well as the DFT‐optimised structure of Fe^3+^/**H**.

## Conflict of interest

There are no conflicts of interest to declare.

1

## Supporting information

As a service to our authors and readers, this journal provides supporting information supplied by the authors. Such materials are peer reviewed and may be re‐organized for online delivery, but are not copy‐edited or typeset. Technical support issues arising from supporting information (other than missing files) should be addressed to the authors.

Supporting Information

## Data Availability

The data that support the findings of this study are available in the supplementary material of this article.
