# Supplementary material for: Selective Fluorescent Sensing for Iron in Aqueous Solution by A Novel Functionalized Pillar[5]arene
Source: ChemistryOpen. 2023 Oct 6;12(10):e202300109. doi: 10.1002/open.202300109 (PMC10558425; doi:10.1002/open.202300109)
Supplement: Supplementary file 1 — Supporting Information [file OPEN-12-e202300109-s001.pdf]

ChemistryOpen

Supporting Information

Selective Fluorescent Sensing for Iron in Aqueous Solution by A Novel Functionalized Pillar[5]arene

Yahan Zhang, Longming Chen, Xinbei Du, Xiang Yu, Han Zhang, Zhao Meng, Zhibing Zheng,* Junyi Chen,* and Qingbin Meng*

Table of Contents

1. General materials and methods 1

1.1 Materials 1

1.2 Instruments 1

2. Synthetic Protocols of water-soluble functionalized pillar[n]arene 2

2.1 Synthesis of Qui-OEPns 2

2.2 Synthesis of compound 2 4

2.3 Synthesis of compound 3 5

2.4 Synthesis of **H** 6

2.5 General procedure for fluorescence experiments 7

3. Supporting results and experimental raw data 8

3.1 ^{1}H NMR ^{13}C NMR and MALDI-TOF-MS spectra of Qui-OEPns 8

3.2 ^{1}H NMR and MALDI-TOF-MS spectra of compound 2 18

3.3 ^{1}H NMR ^{13}C NMR and MALDI-TOF-MS spectra of compound 3 19

3.4 ^{1}H NMR ^{13}C NMR and MALDI-TOF-MS spectra of **H** 21

3.5 UV-visible spectroscopy of **H** upon addition of Fe^{3+} 23

3.6 ^{1}H NMR spectra **H** in the presence of increasing concentration of Fe^{3+} 23

3.7 ^{1}H NMR spectrum of TEP5 with addition of Fe^{3+} 24

3.8 UV-visible spectroscopy of **H** upon addition of Fe^{3+} 24

3.9 Job's plot analysis for **H** with Fe^{3+} 25

3.10 Optimized geometries of Fe^{3+} with **H** 25

3.11 Fluorescence spectra of **H** with Fe^{3+} in water 26

3.12 Calibration curves of Fe^{3+} 26

3.13 Practical application of **H** for accurate detection of iron concentration 27

1. General materials and methods

1.1 Materials. All solvents and reagents were purchased commercially and used without further purification. $FeCl_3$, $ZnCl_2$, $MgCl_2$, $BaCl_2$, $AlCl_3$, $NiCl_2$, $CoCl_2$, $CuCl_2$, $CdCl_2$, $CrCl_3$, $MnCl_2$, KCl and NaCl were purchased from Energy Chemical. Iron Assay Kit was purchased by Adsbio (Jiangsu, China).

1.2 Instruments. The ^{1}H- and ^{13}C-NMR spectra were recorded at Bruker Advance 600 MHz. Molecular weights were analyzed by matrix-assisted laser desorption/ionization time-of-flight mass spectrometry (MALDI-TOF-MS; Bruker Reex, Bruker Daltonic, Inc., CA, USA). Fluorescence spectroscopic studies were recorded using a LS-55 fluorescence spectrophotometer Perkin Elmer Co.Ltd.

2. Synthetic Protocols of water-soluble functionalized pillar[n]arene

2.1 Synthesis of Qui-OEPns

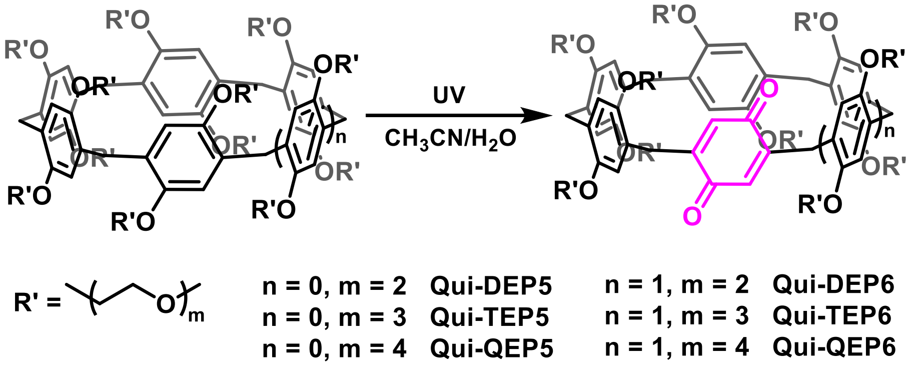

Scheme S1. Synthesis of di-hydroxylated pillar[n]arene bearing with oligoethylene oxide substituents (Qui-OEPns)

Synthesis of pillar[n]arene modified with oligoethylene oxide group (OEPns): Pillar[n]arene modified with oligoethylene oxide group were synthesized according to the previous paper.[1]

Synthesis of Qui-OEPns: In brief, Qui-TEP5 for example, pillar[5]arenes modified with triethylene oxide group (TEP5) (1 mmol) was added in 6 mL mixed-solvent of acetonitrile and water (1:1), The mixture was stirred under UV (303 nm) exposure at room temperature for 10 hours. The obtained crude product was concentrated under vacuum. The residue was purified by column chromatography on silica gel to afford Qui-TEP5 (0.62g, 35%). Qui-DEP5 (0.67g, 47%), Qui-QEP5 (0.70g, 33%), Qui-DEP6 (0.74g, 42%), Qui-TEP6 (0.50g, 23%), Qui-QEP6 (0.55g, 21%) were also afforded according to the same method.

Qui-DEP5: Red liquid; ^{1}H NMR ($CDCl_3$, 600 MHz, ppm): δ 6.84, 6.82, 6.80, 6.74, 6.68 (s, 10H), 3.96-4.06, 3.54-3.87 (m, 74H), 3.35-3.31 (m, 24H). ^{13}C NMR ($CDCl_3$, 150 MHz, ppm): δ188.73, 150.39, 150.00, 149.70, 146.09, 133.56, 130.07, 128.85, 128.75, 123.95, 115.71, 114.92, 72.09, 71.87, 70.69, 70.58, 70.21, 69.87, 68.25, 67.98, 67.47, 59.03, 29.75, 27.75. MALDI-TOF-MS (m/z): $[M+H]^+$ calcd. for $C_{75}H_{109}O_{26}$, 1425.721, found 1426.506.

Qui-TEP5: Red liquid; ^{1}H NMR ($CDCl_3$, 600 MHz, ppm): δ 6.91, 6.89, 6.84, 6.77, 6.65 (s, 10H), 3.99-4.05, 3.43-3.87 (m, 106H), 3.31-3.28 (m, 24H). ^{13}C NMR ($CDCl_3$,

150 MHz, ppm): δ 188.71, 150.45, 149.97, 149.75, 149.71, 146.09, 133.58, 130.06, 128.86, 128.69, 139.99, 115.67, 114.91, 71.89, 70.82, 70.73, 70.58, 70.49, 70.29, 69.99, 68.26, 67.95, 67.59, 59.00, 29.75, 29.22, 27.75. MALDI-TOF-MS (m/z): $[M+Na]^+$ calcd. for $C_{91}H_{140}NaO_{34}$, 1799.913; found 1799.534.

Qui-QEP5: Red liquid; ^1H NMR ($CDCl_3$, 600 MHz, ppm): δ 6.87, 6.85, 6.83, 6.76, 6.65 (s, 10H), 4.07-3.96, 3.90-3.46 (m, 138H) 3.36-3.26 (m, 24H). ^{13}C NMR ($CDCl_3$, 150 MHz, ppm): δ 188.68, 150.44, 150.02, 149.74, 146.17, 133.56, 130.04, 128.84, 128.61, 124.07, 115.63, 114.93, 71.91, 70.76, 70.68, 70.61, 70.54, 70.45, 70.43, 70.26, 70.23, 70.10, 70.02, 68.25, 67.94, 67.60, 59.03, 31.95, 29.76, 29.26. MALDI-TOF-MS (m/z): $[M+Na]^+$ calcd. for $C_{107}H_{172}NaO_{42}$, 2152.122; found 2151.749.

Qui-DEP6: Red liquid; ^1H NMR ($CDCl_3$, 600 MHz, ppm): δ 6.77, 6.71, 6.67, 6.64, 6.46 (s, 12H), 4.05-3.88, 3.83-3.48 (m, 92H), 3.39-3.34 (m, 30H). ^{13}C NMR ($CDCl_3$, 150 MHz, ppm): δ 188.33, 150.86, 150.85, 146.18, 133.94, 129.52, 128.50, 128.28, 127.69, 123.32, 115.99, 115.73, 115.62, 115.45, 115.08, 72.07, 72.01, 70.72, 70.61, 70.10, 70.05, 69.92, 68.52, 68.41, 68.33, 59.08, 31.20, 30.59, 29.76. MALDI-TOF-MS (m/z): $[M+Na]^+$ calcd. for $C_{92}H_{134}NaO_{32}$, 1773.876; found 1775.499.

Qui-TEP6: Red liquid; ^1H NMR ($CDCl_3$, 600 MHz, ppm): δ 6.77, 6.70, 6.65, 6.63, 6.47 (s, 12H), 4.02-3.87, 3.83-3.49 (m, 132H), 3.37-3.32 (m, 30H). ^{13}C NMR ($CDCl_3$, 150 MHz, ppm): δ 188.31, 150.89, 150.82, 150.67, 150.58, 146.24, 133.93, 129.59, 128.52, 127.82, 123.46, 116.10, 115.88, 115.70, 115.56, 115.24, 71.93, 70.73, 70.65, 70.60, 70.52, 70.04, 69.87, 68.59, 68.53, 68.47, 68.36, 59.01, 31.94, 31.20, 30.51, 29.76. MALDI-TOF-MS (m/z): $[M+Na]^+$ calcd. for $C_{112}H_{174}NaO_{42}$, 2214.138; found 2213.804.

Qui-QEP6: Red liquid; ^1H NMR ($CDCl_3$, 600 MHz, ppm): δ 6.76, 6.69, 6.65, 6.63, 6.46 (s, 12H), 4.02-3.87, 3.82-3.50, (m, 172H), 3.39-3.34 (m, 30H). ^{13}C NMR ($CDCl_3$, 150 MHz, ppm): δ 152.58, 136.76, 130.39, 129.90, 129.33, 127.67, 121.05, 116.05, 71.92, 70.50, 68.62, 66.68, 66.37, 63.68, 62.68, 62.12, 61.72, 60.88, 59.03, 31.97, 31.30, 30.58, 29.75. MALDI-TOF-MS (m/z): $[M+Na]^+$ calcd. for $C_{132}H_{214}NaO_{52}$, 2654.400; found 2654.740.

2.2 Synthesis of compound 2

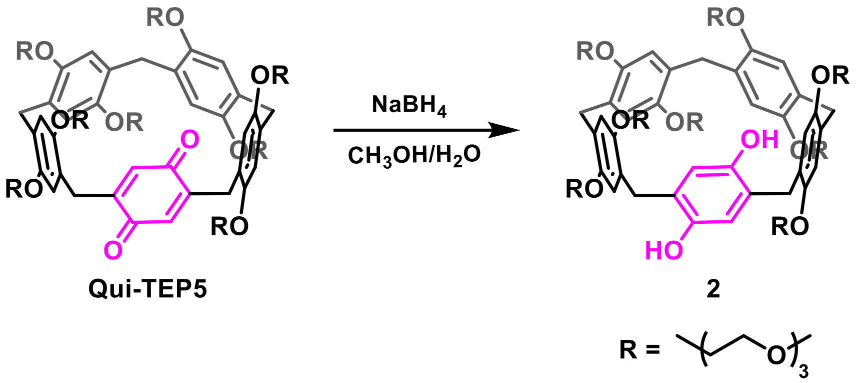

Synthesis of compound 2: To a solution of Qui-TEP5 (1.00 g, 0.56 mmol) in methanol (20 mL) and water (20 mL), $NaBH_4$ (101 mg, 2.35 mmol) was added. The mixture was stirred at 25 °C for 2h. The reaction was quenched by pouring into 1 M HCl aqueous solution. After evaporating the solvents, the mixture was extracted with dichloromethane. After filtration, solvents were evaporated to give a brown liquid (0.94 g, 0.53 mmol, 94%). ^1H NMR ($CDCl_3$, 600 MHz, ppm): δ 6.92, 6.82, 6.81, 6.63, 6.54 (s, 10H, phenyl and benzoquinone), 4.11-3.97, 3.88-3.43 (m, 106H, methylene), 3.32-3.25 (m, 24H, methyl). MALDI-TOF-MS (m/z): $[M+Na]^+$ calcd. for $C_{91}H_{142}NaO_{34}$, 1801.928; found 1801.248.

2.3 Synthesis of compound 3

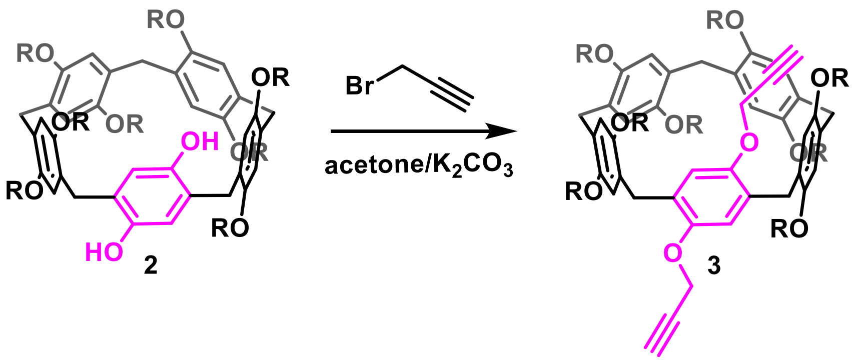

Synthesis of compound 3: Under a nitrogen atmosphere, to a solution of compound 2 (800 mg, 0.45 mmol) in acetone (50.0 mL), K_2CO_3 (373 mg, 2.70 mmol) and 3-bromopropyne (321.19 mg, 2.70 mmol) were added. The resulting mixture was stirred at reflux overnight and the reaction was stopped by filtration and evaporated under vacuum to get a brown liquid (778 mg, 0.42mmol, 93%). ^{1}H NMR ($CDCl_3$, 600 MHz, ppm): δ 6.86, 6.82, 6.81, 6.79, 6.74 (s, 10H, phenyl and benzoquinone), 4.52 (d, 4H), 3.99-3.45, (m, 106H), 3.29-3.27 (m, 24H), 2.23 (s, 2H). ^{13}C NMR ($CDCl_3$, 150 MHz, ppm): δ 149.99, 149.96, 149.79, 149.73, 149.29, 129.12, 128.91, 128.60, 128.41, 115.63, 115.45, 115.22, 115.00, 71.80, 70.79, 70.70, 70.66, 70.46, 70.29, 70.18, 70.15, 70.12, 58.91, 56.43, 29.69, 29.57, 29.32. MALDI-TOF-MS (m/z): $[M+Na]^+$ calcd. for Chemical Formula: $C_{97}H_{146}NaO_{34}$, Molecular Weight: 1877.960, found 1877.422.

2.4 Synthesis of H

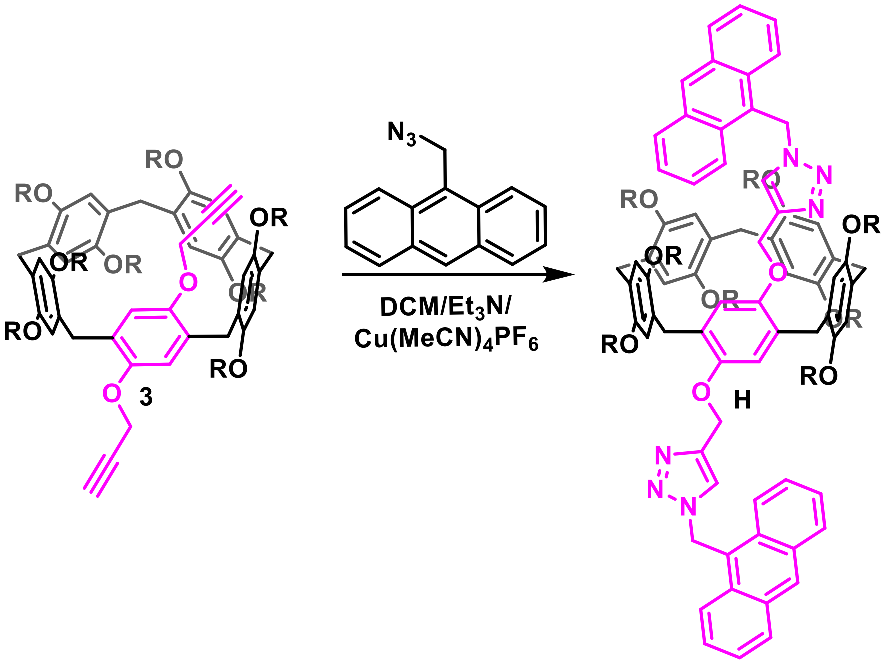

Synthesis of H: A mixture of compound 3 (250 mg, 0.13 mmol), 9-azidomethylanthracene (90.9 mg, 0.39 mmol), $Cu(MeCN)_4PF_6$ (145.1 mg, 0.39 mmol), and triethylamine (39.4 mg, 0.39 mmol) in dichloromethane (50 ml) was stirred under N_2 atmosphere for 24 h. The solvent was removed by a rotary evaporator and the residue was purified by reverse-phase high-performance liquid chromatography (HPLC) using a C8 column (Waters, USA) and a gradient of acetonitrile and deionized water containing 0.1% TFA. The solvent evaporated via lyophilization process to get brown liquid (220 mg, 0.095 mmol, 73%). ^{1}H NMR (CD_3CN, 600 MHz, ppm): δ 8.65 (d, 2H), 8.51 (m, 4H), 8.13 (m, 4H), 7.74 (s, 2H), 7.63 (m, 4H), 7.54 (m, 4H), 6.92 (m, 4H), 6.87 (s, 4H), 6.66 (s, 2H), 6.60 (s, 4H), 4.90-4.84 (d, 4H), 3.98-3.40 (m, 106H), 3.22-3.11 (m, 24H). ^{13}C NMR (CD_3CN, 150 MHz, ppm): δ 150.18, 150.06, 150.00, 149.88, 144.77, 132.24, 131.38, 130.03, 128.06, 126.17, 124.30, 116.35, 115.28, 115.14, 114.89, 72.31, 72.16, 71.17, 71.08, 70.99, 70.87, 70.76, 70.72, 70.57, 70.48, 70.29, 68.70, 68.66, 68.61, 68.45, 62.82, 58.62, 58.50, 46.78, 31.30, 30.21, 30.09, 29.49, 29.35. MALDI-TOF-MS (m/z): $[M+Na]^+$ calcd. for Chemical Formula: $C_{127}H_{168}NaN_6O_{34,}$ Molecular Weight: 2344.150, found 2345.464.

2.5 General procedure for fluorescence experiments

The solution of **H** (20 μM) was prepared with pure water. The chloride salts of various ions were dissolved in the solution **H**. During the fluorescence experiments the concentration of **H** was kept at 20 μM and the concentration of metal salts was increased gradually according to previously reported method.[2]

3. Supporting results and experimental raw data

3.1 ^{1}H NMR ^{13}C NMR and MALDI-TOF-MS spectra of Qui-OEPns

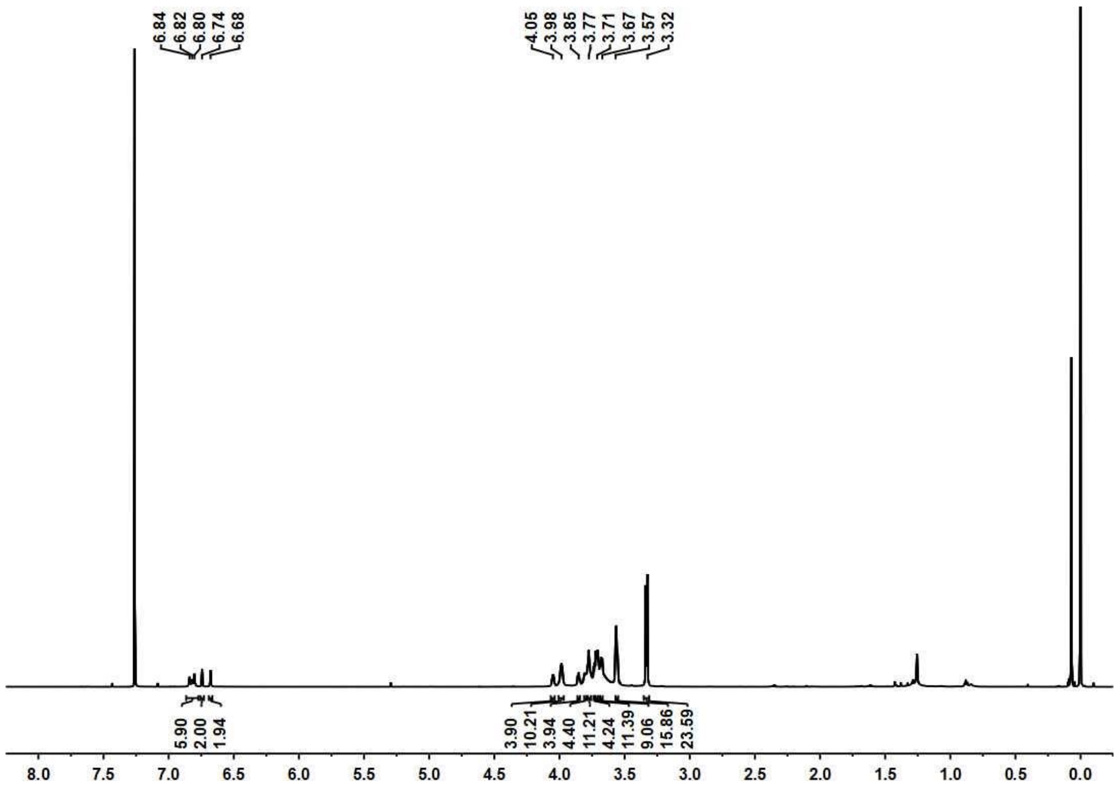

Figure S1. ^{1}H NMR spectrum (600 MHz, $CDCl_3$, 298K) of Qui-DEP5.

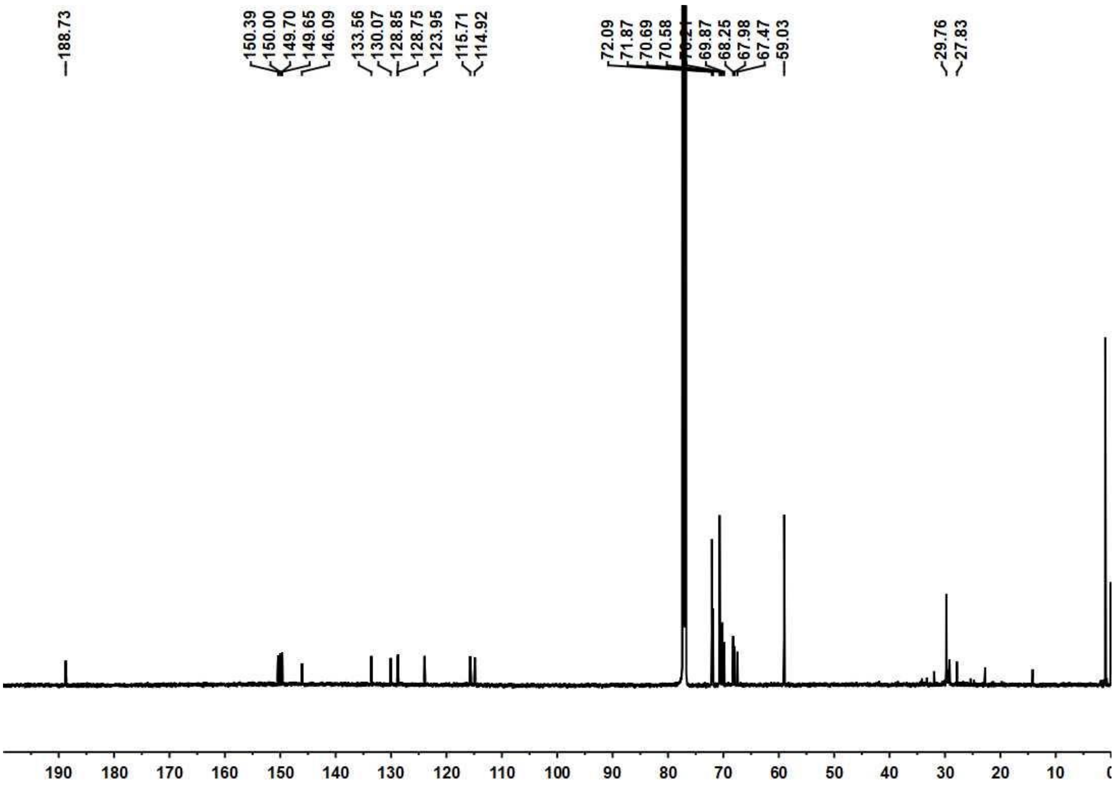

Figure S2. ^{13}C NMR spectrum (150 MHz, $CDCl_3$, 298K) of Qui-DEP5.

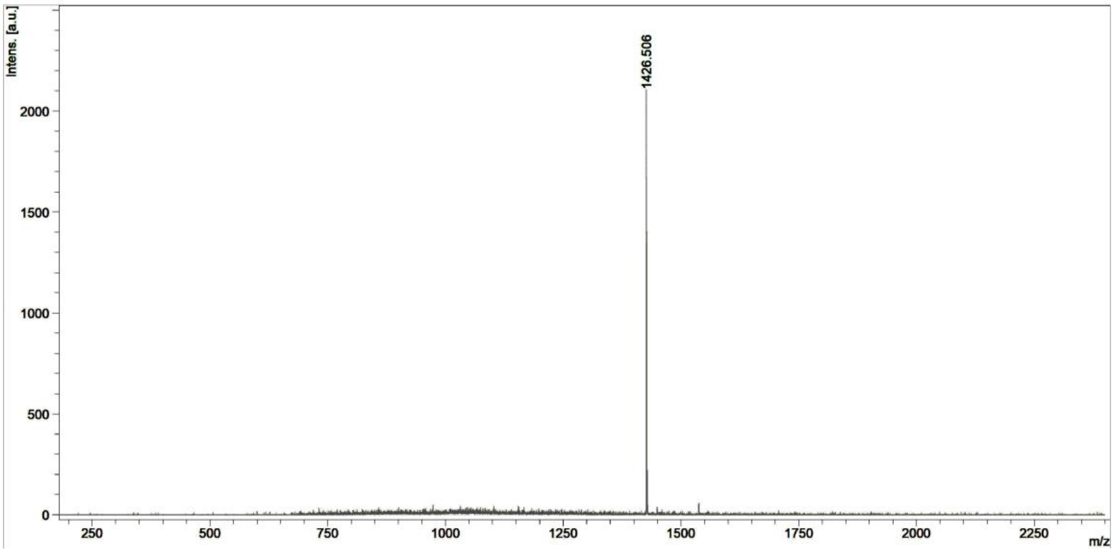

Figure S3. MALDI-TOF-MS of Qui-DEP5.

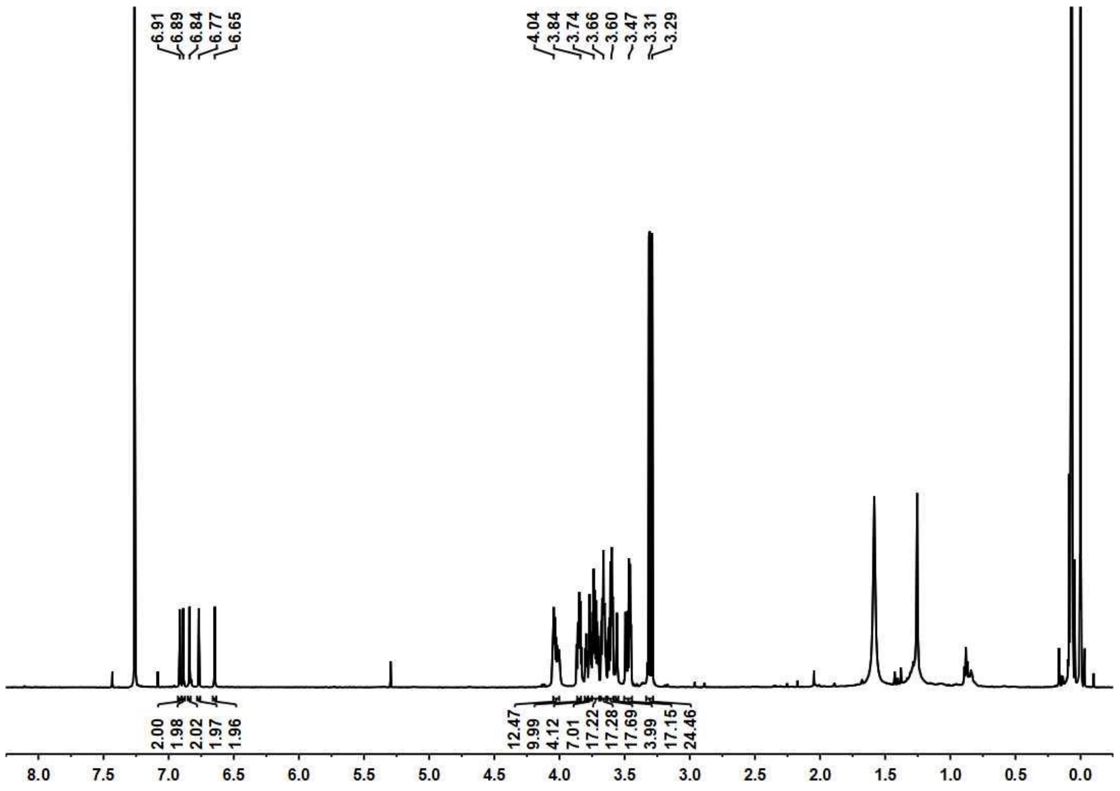

Figure S4. ^{1}H NMR spectrum (600 MHz, $CDCl_3$) of Qui-TEP5.

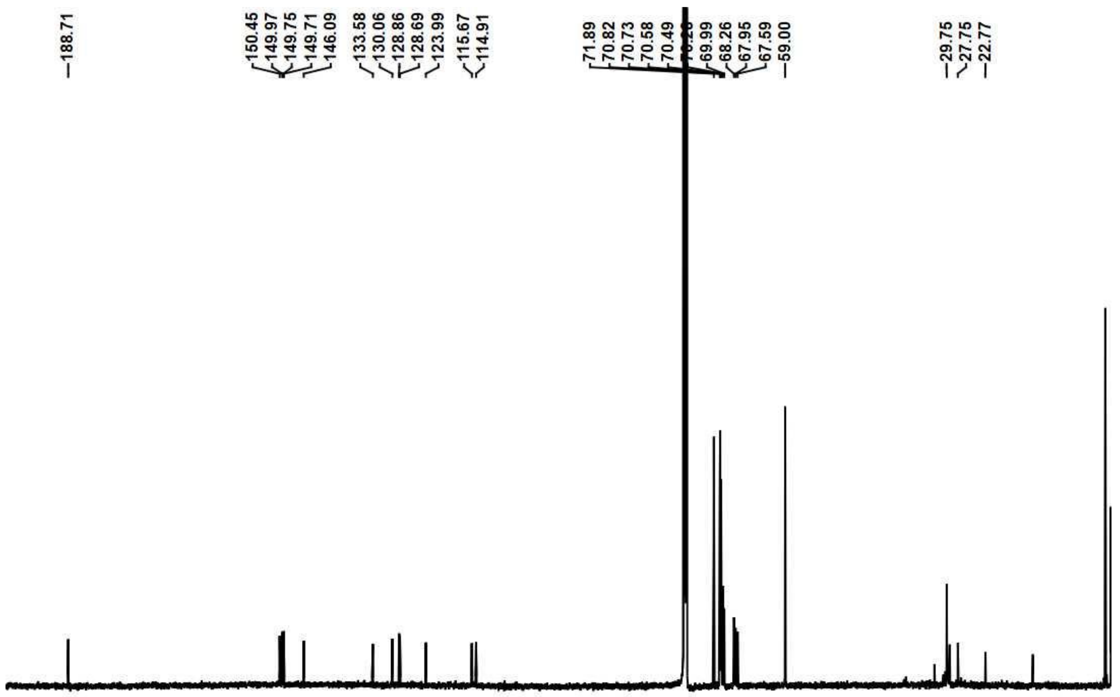

Figure S5. ^{13}C NMR spectrum (150 MHz, $CDCl_3$, 298K) of Qui-TEP5.

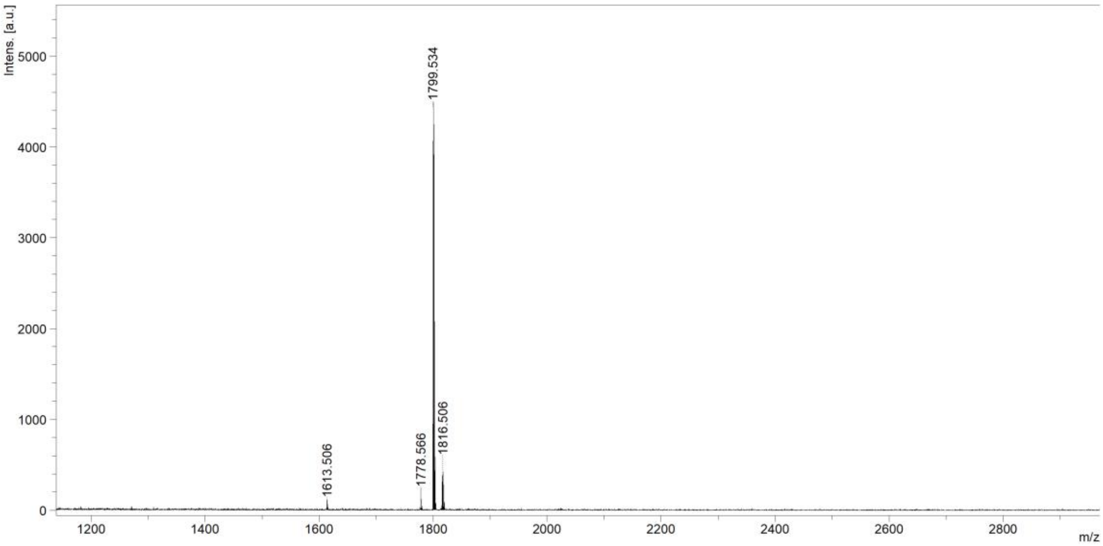

Figure S6. MALDI-TOF-MS of Qui-TEP5.

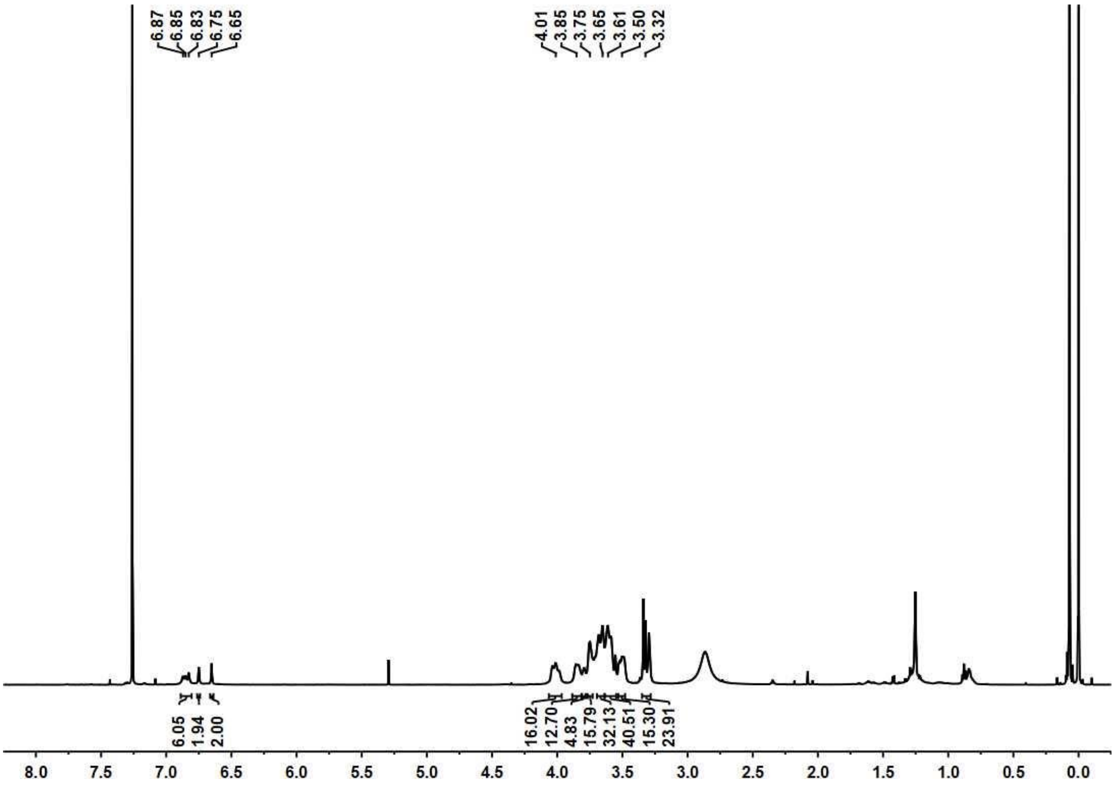

Figure S7. ^{1}H NMR spectrum (600 MHz, $CDCl_3$, 298K) of Qui-QEP5.

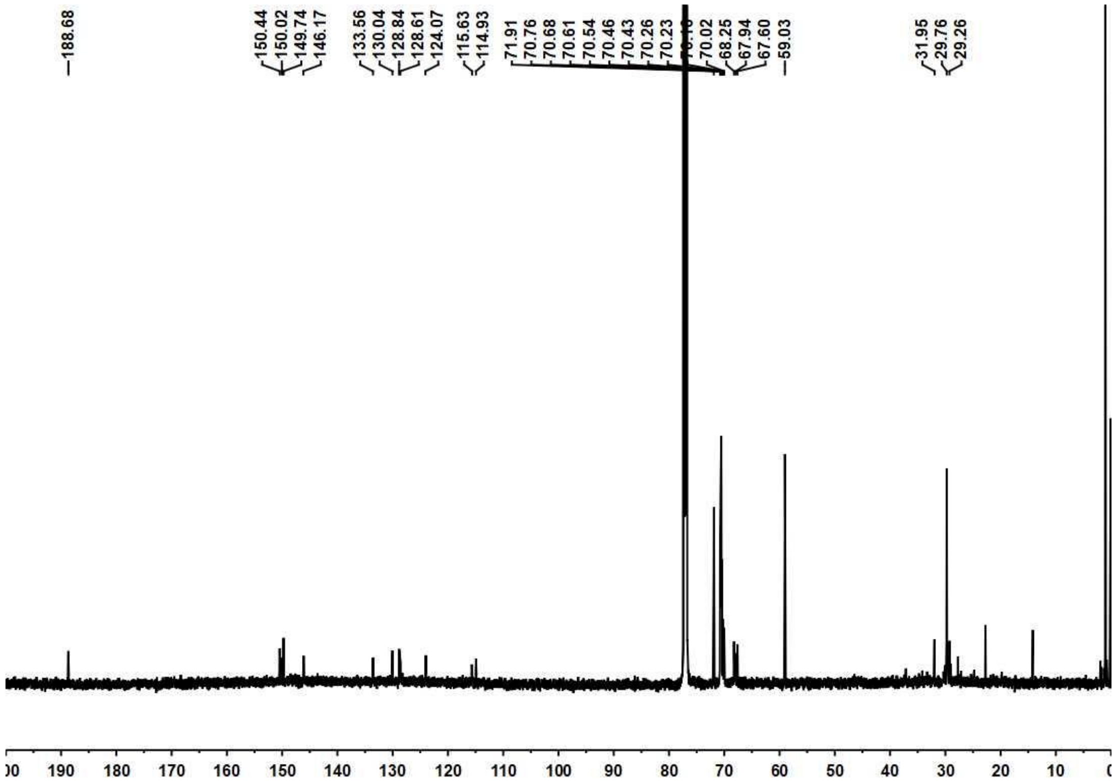

Figure S8. ^{13}C NMR spectrum (150 MHz, $CDCl_3$, 298K) of Qui-QEP5.

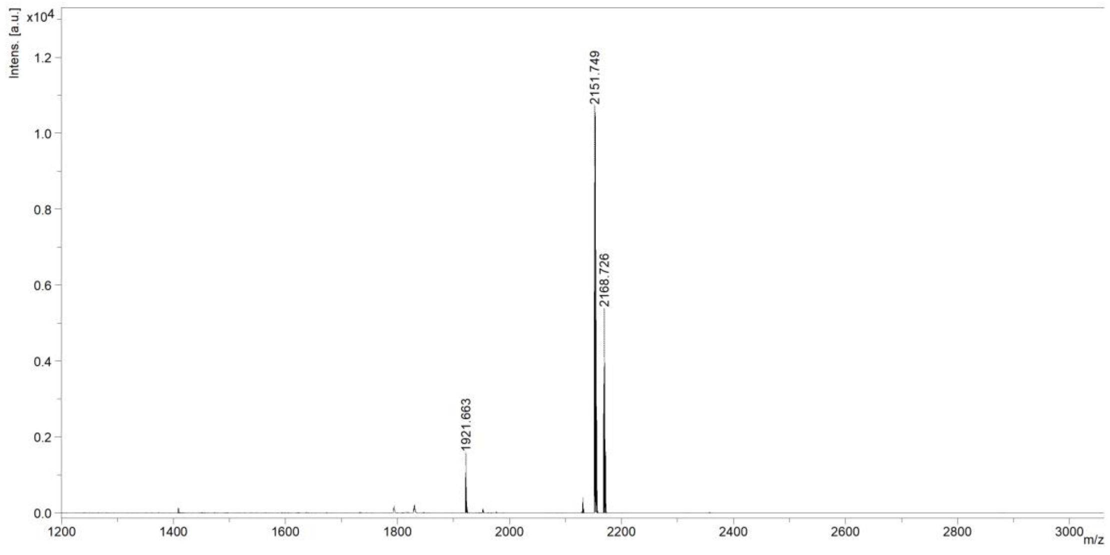

Figure S9. MALDI-TOF-MS of Qui-QEP5.

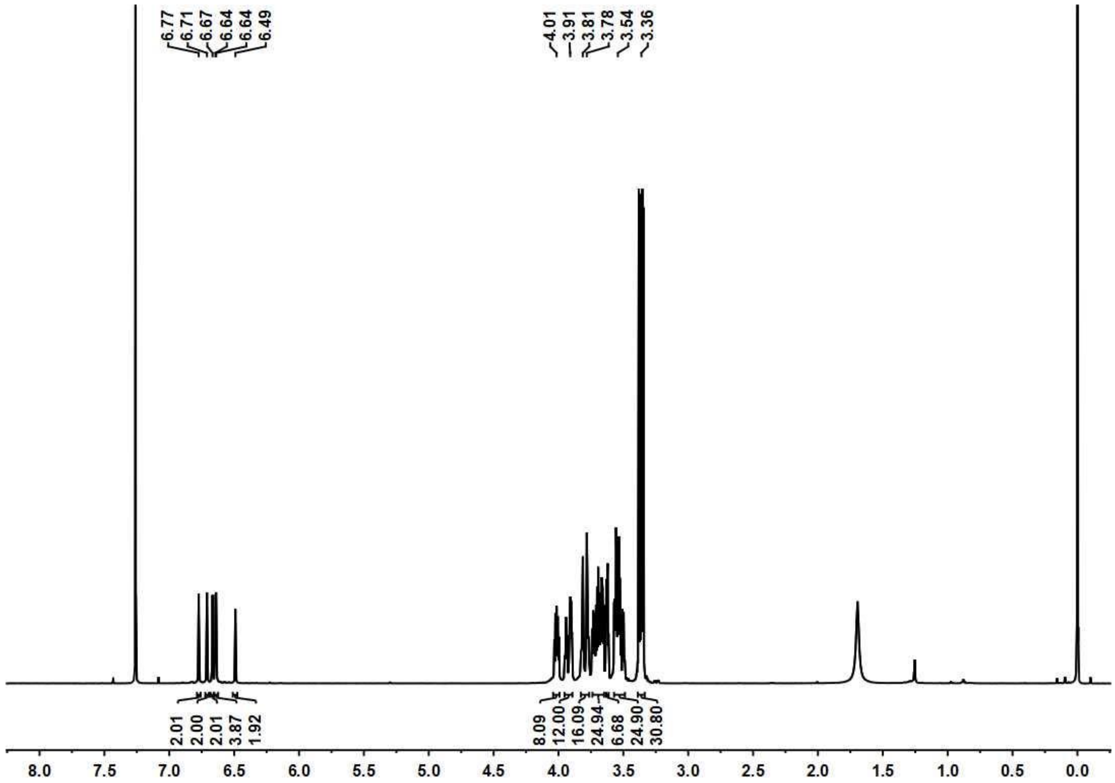

Figure S10. ^{1}H NMR spectrum (600 MHz, $CDCl_3$, 298K) of Qui-DEP6.

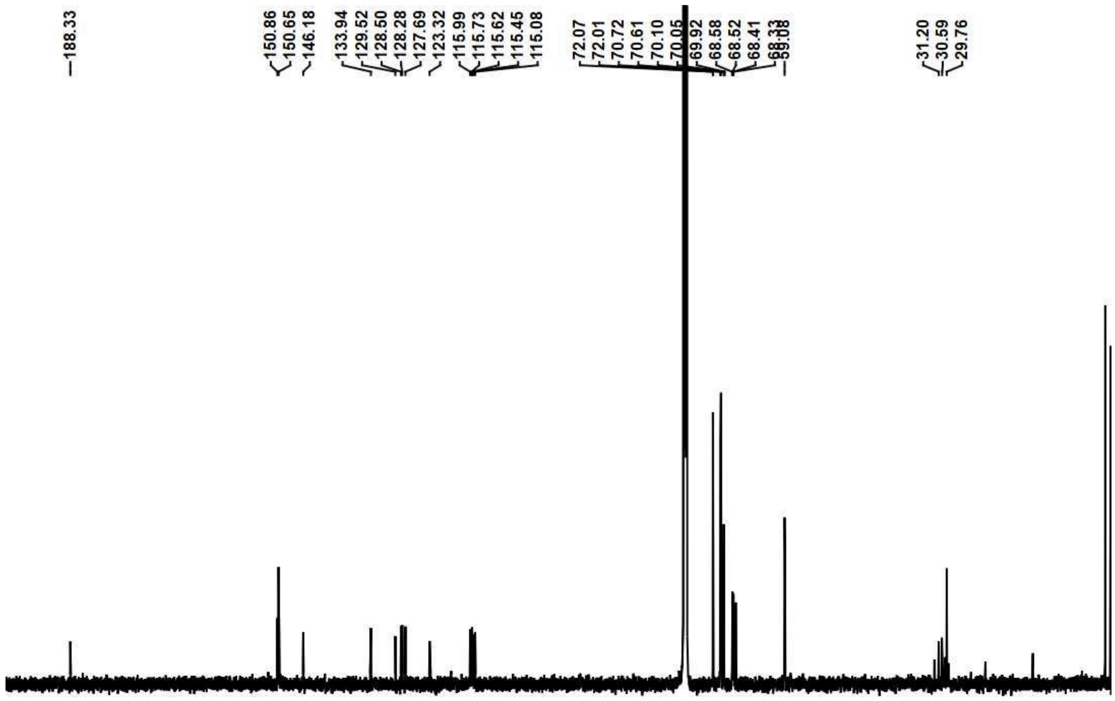

Figure S11. ^{13}C NMR spectrum (150 MHz, $CDCl_3$, 298K) of Qui-DEP6.

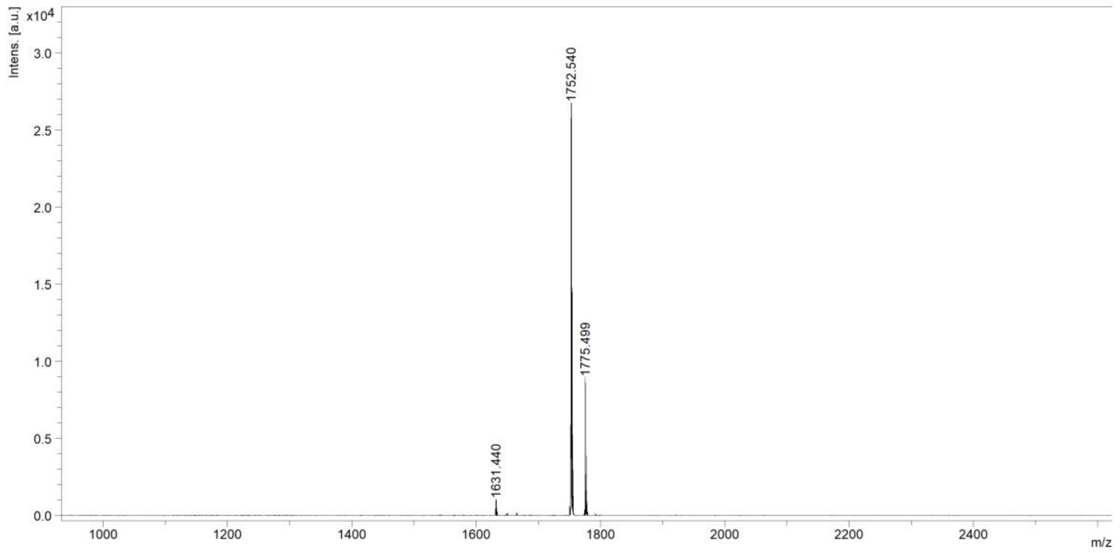

Figure S12. MALDI-TOF-MS of Qui-DEP6.

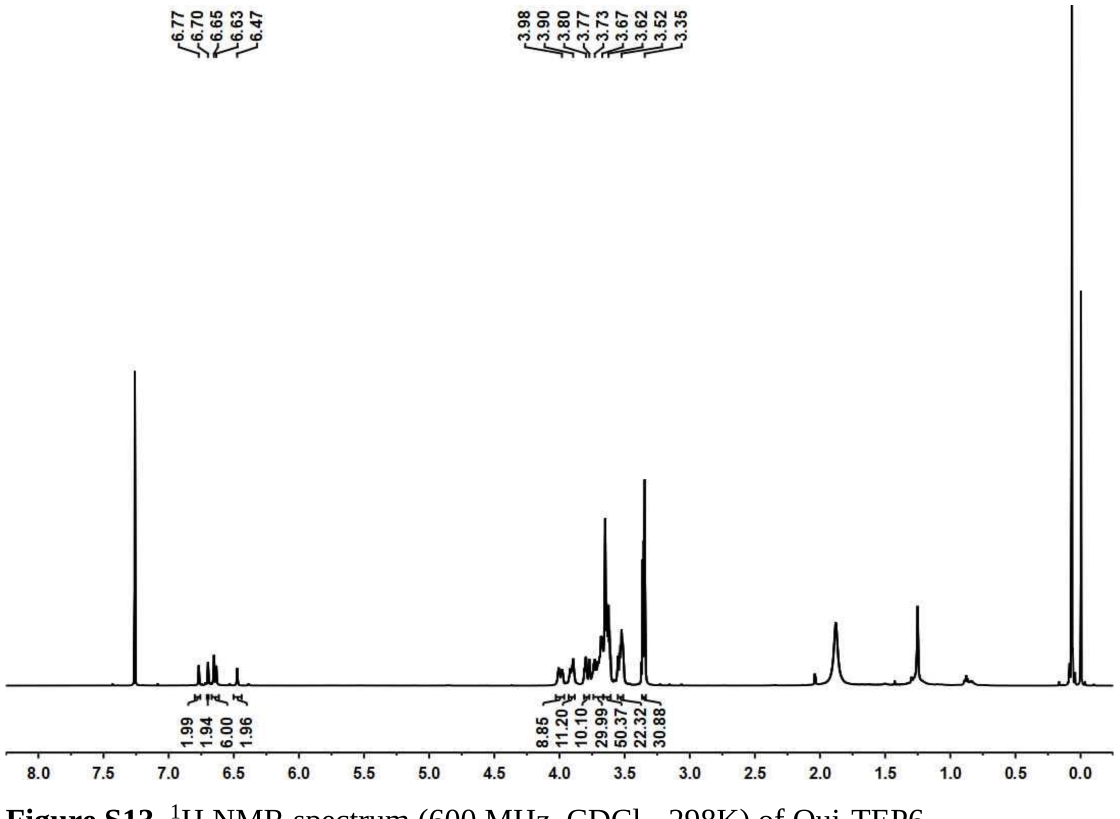

Figure S13. ^{1}H NMR spectrum (600 MHz, $CDCl_3$, 298K) of Qui-TEP6.

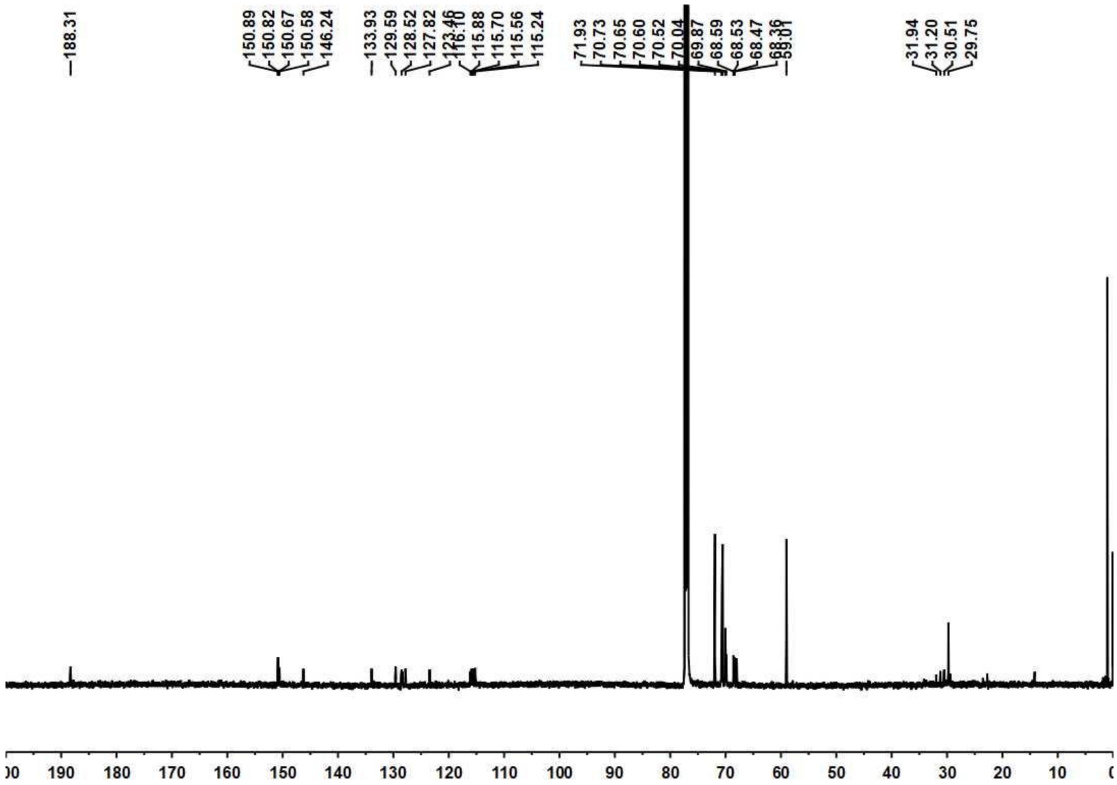

Figure S14. ^{13}C NMR spectrum (150 MHz, $CDCl_3$, 298K) of Qui-TEP6.

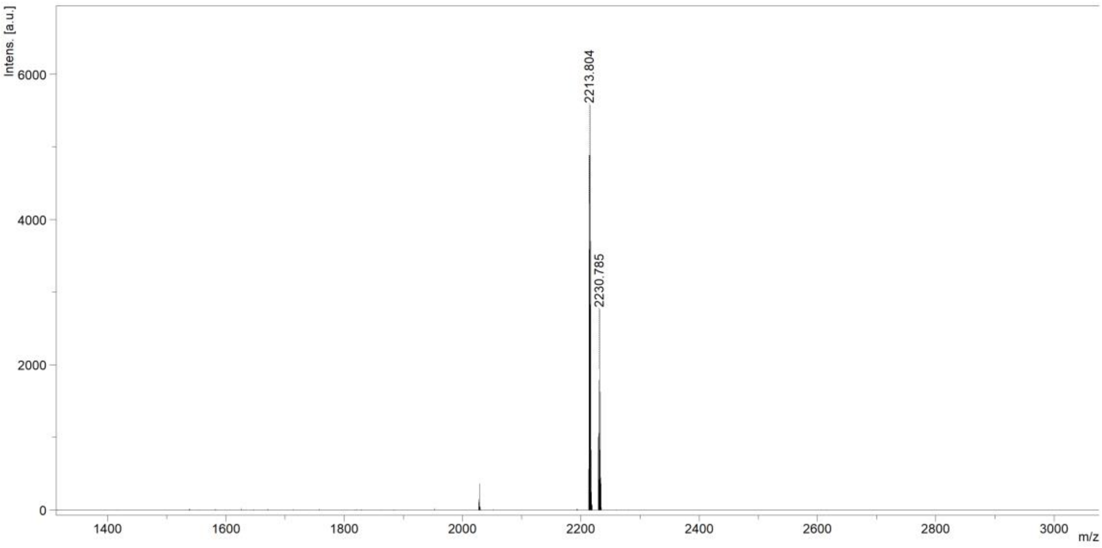

Figure S15. MALDI-TOF-MS of Qui-TEP6.

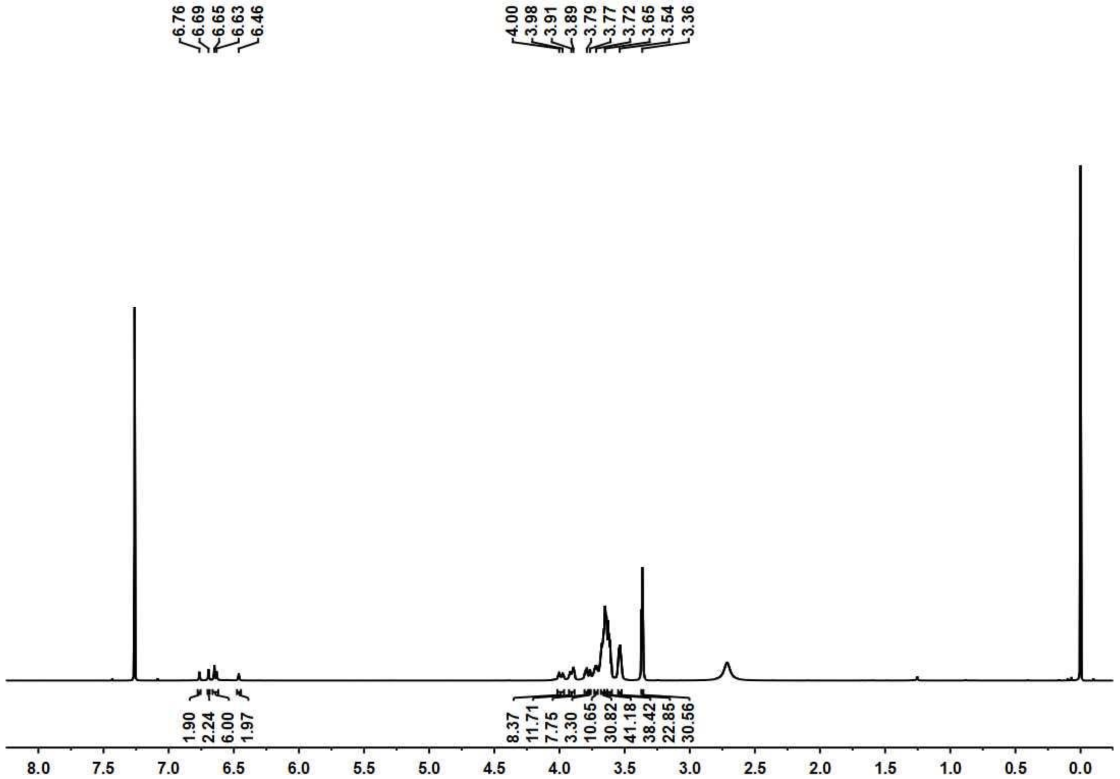

Figure S16. ^{1}H NMR spectrum (600 MHz, $CDCl_3$, 298K) of Qui-QEP6.

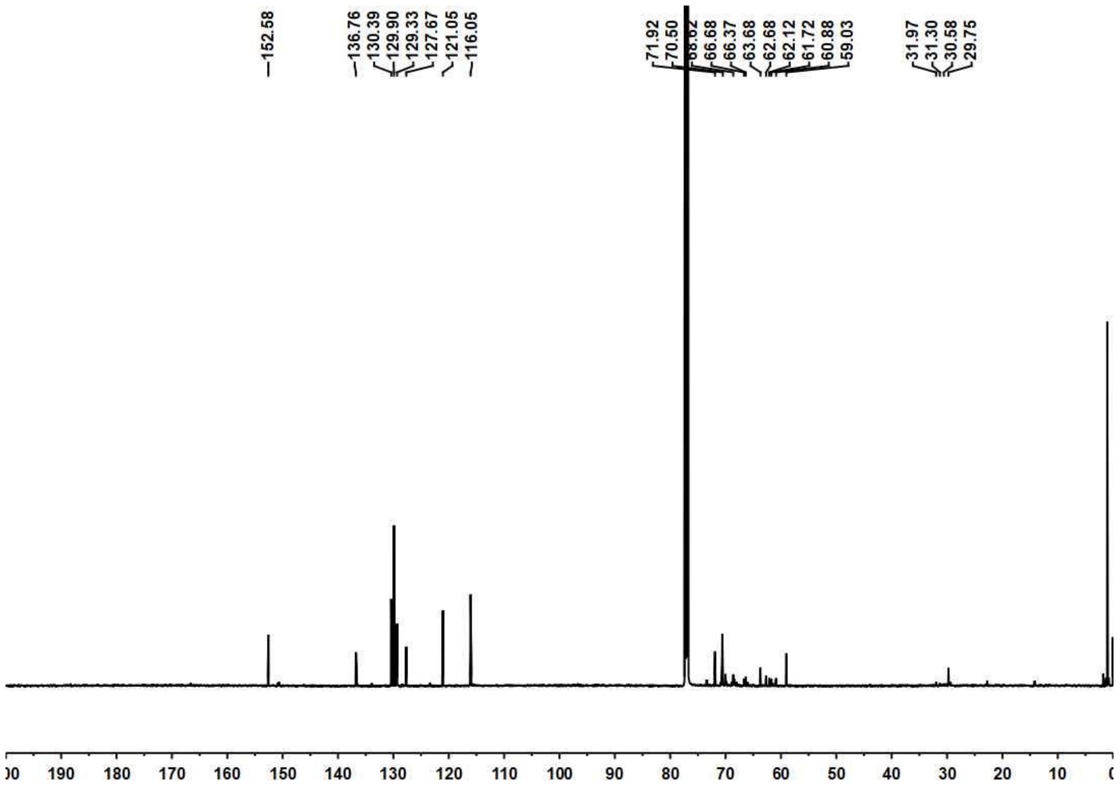

Figure S17. ^{13}C NMR spectrum (150 MHz, $CDCl_3$, 298K) of Qui-QEP6.

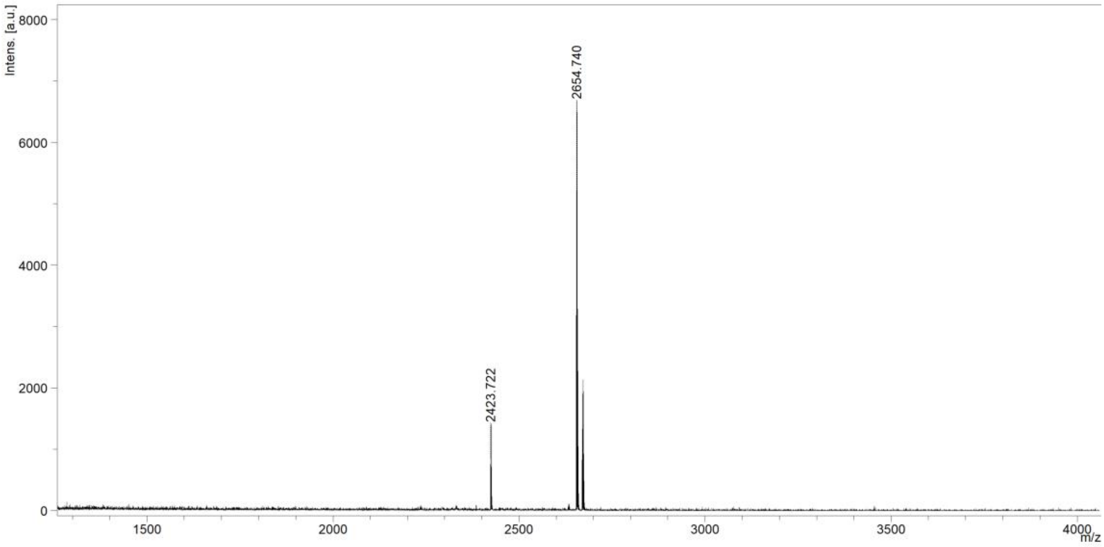

Figure S18. MALDI-TOF-MS of Qui-QEP6.

3.2 ^{1}H NMR and MALDI-TOF-MS spectra of compound 2

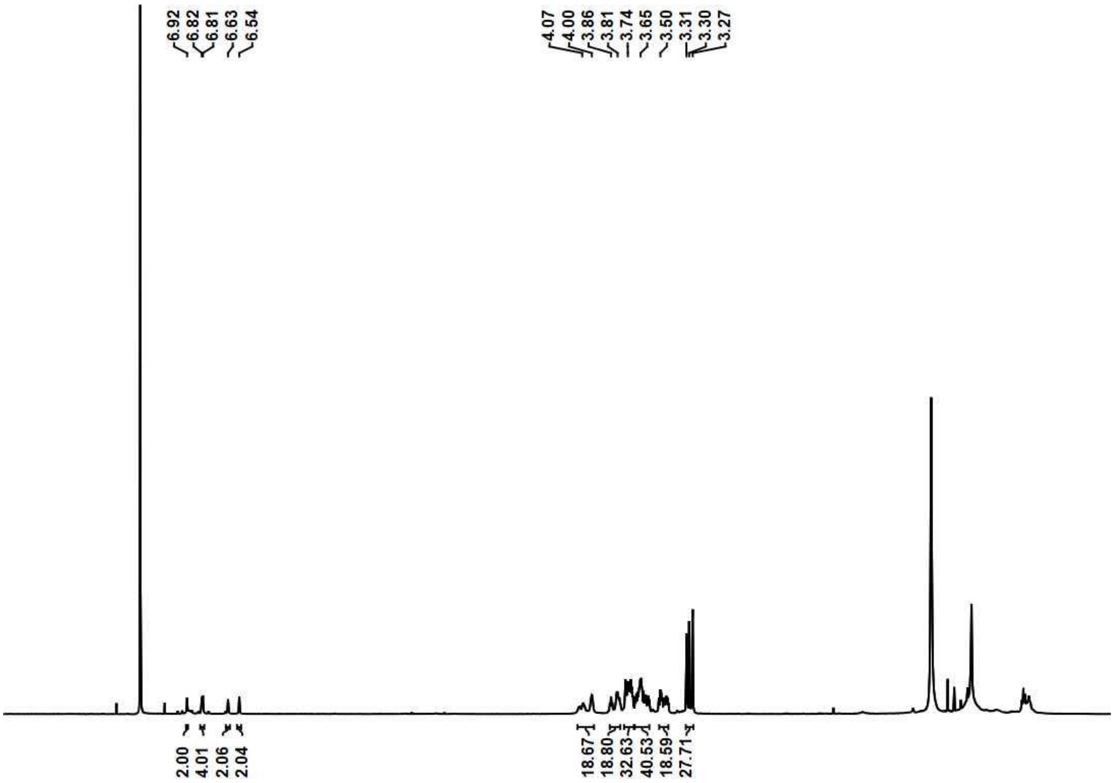

Figure S19. ^{1}H NMR spectrum (600 MHz, $CDCl_3$, 298K) of compound 2.

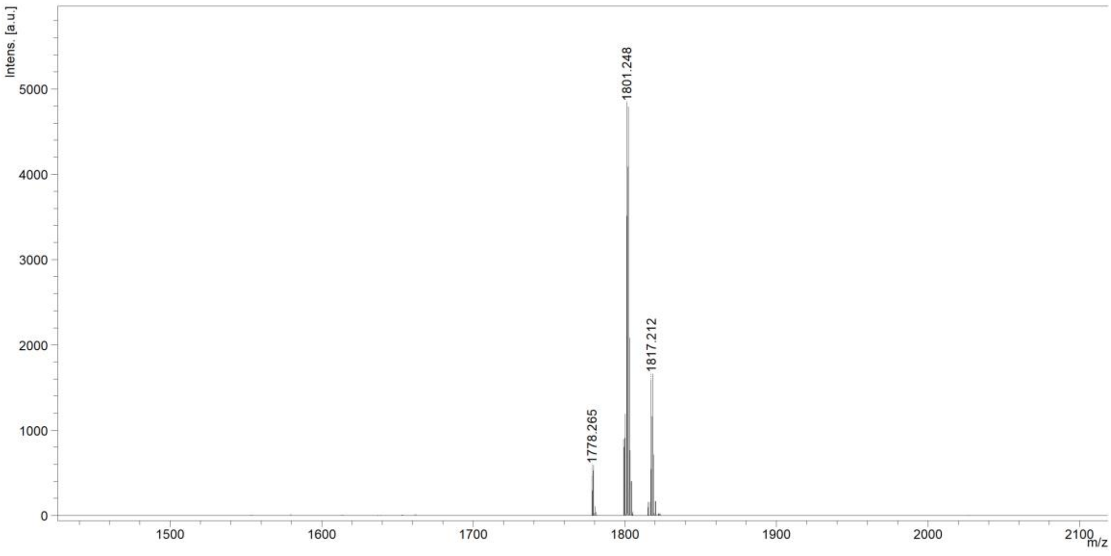

Figure S20. MALDI-TOF-MS of compound 2.

3.3 ^{1}H NMR ^{13}C NMR and MALDI-TOF-MS spectra of compound 3

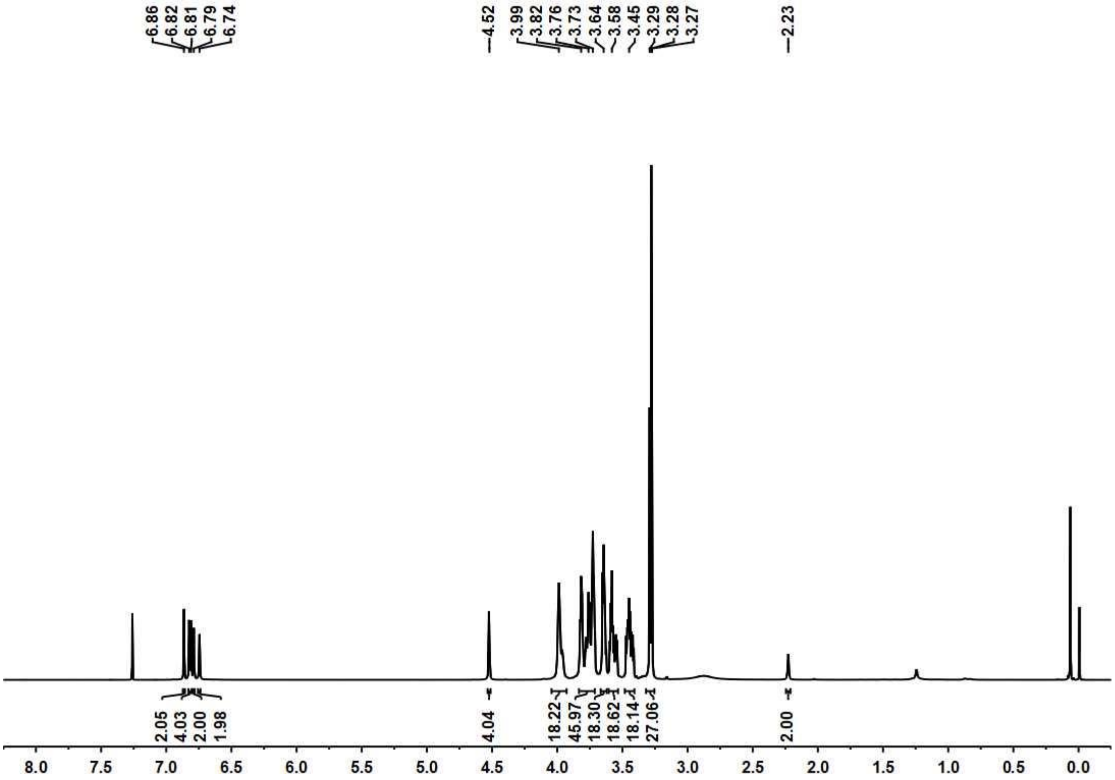

Figure S21. ^{1}H NMR spectrum (600 MHz, $CDCl_3$, 298K) of compound 3.

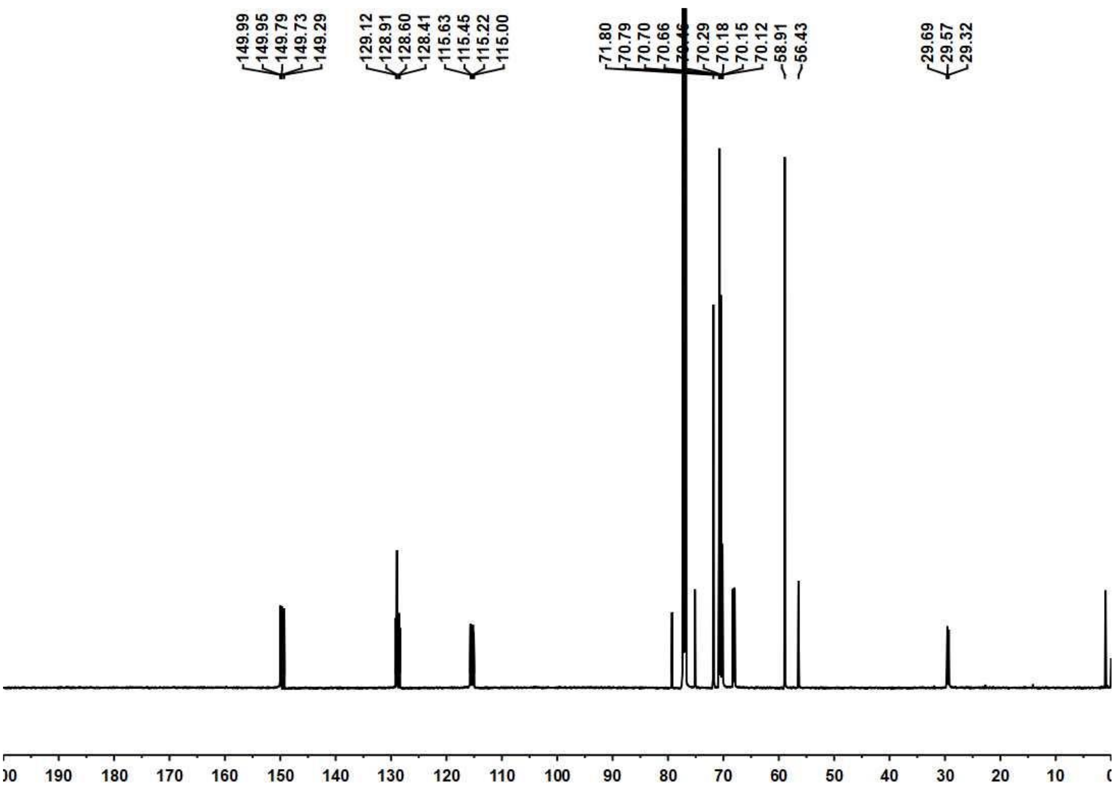

Figure S22. ^{13}C NMR spectrum (150 MHz, $CDCl_3$, 298K) of compound 3.

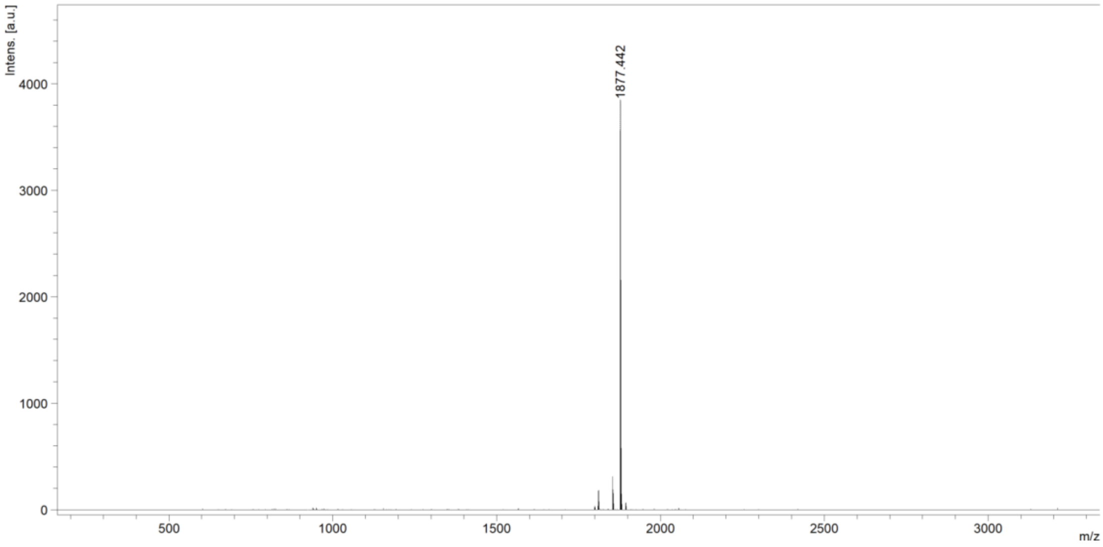

Figure S23. MALDI-TOF-MS of compound 3.

3.4 ^{1}H NMR ^{13}C NMR and MALDI-TOF-MS spectra of H

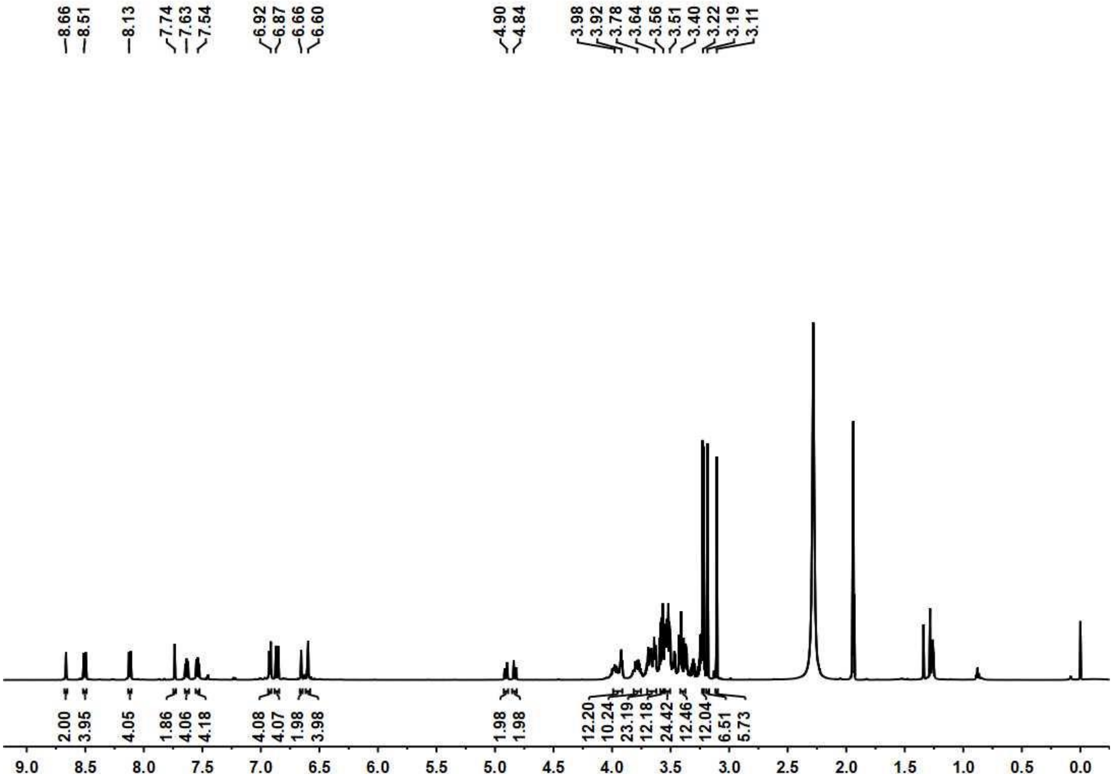

Figure S24. ^{1}H NMR spectrum (600 MHz, CD_3CN, 298K) of **H**.

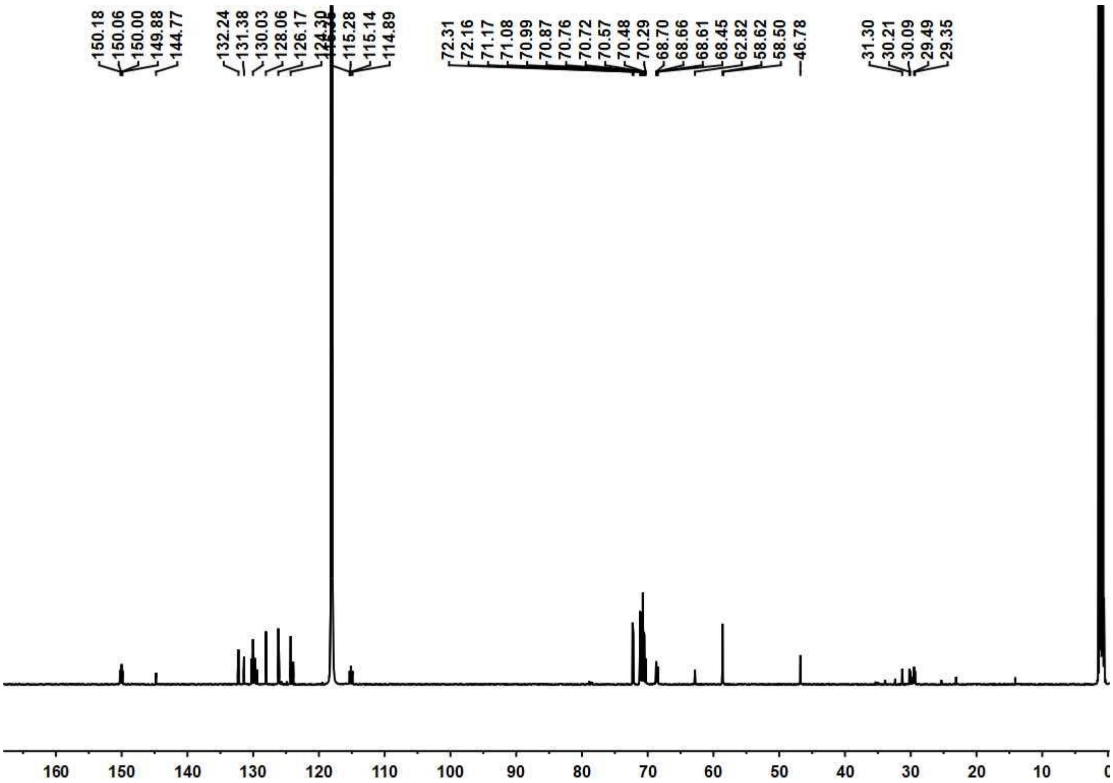

Figure S25. ^{13}C NMR spectrum (150 MHz, CD_3CN, 298K) of **H**.

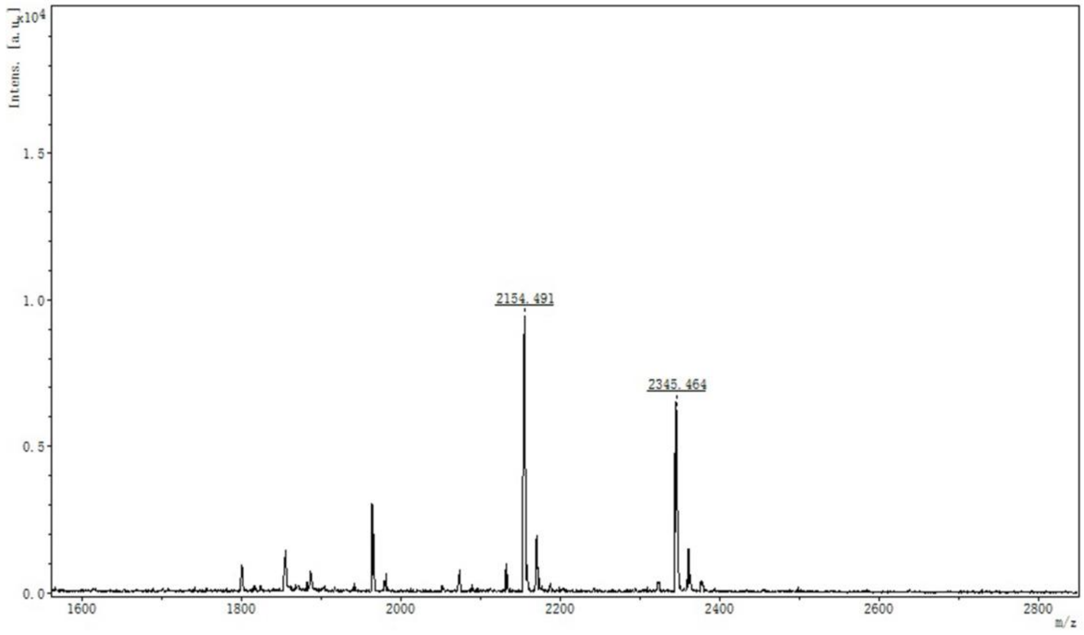

Figure S26. MALDI-TOF-MS of **H**.

3.5 UV-visible spectroscopy of TEP5

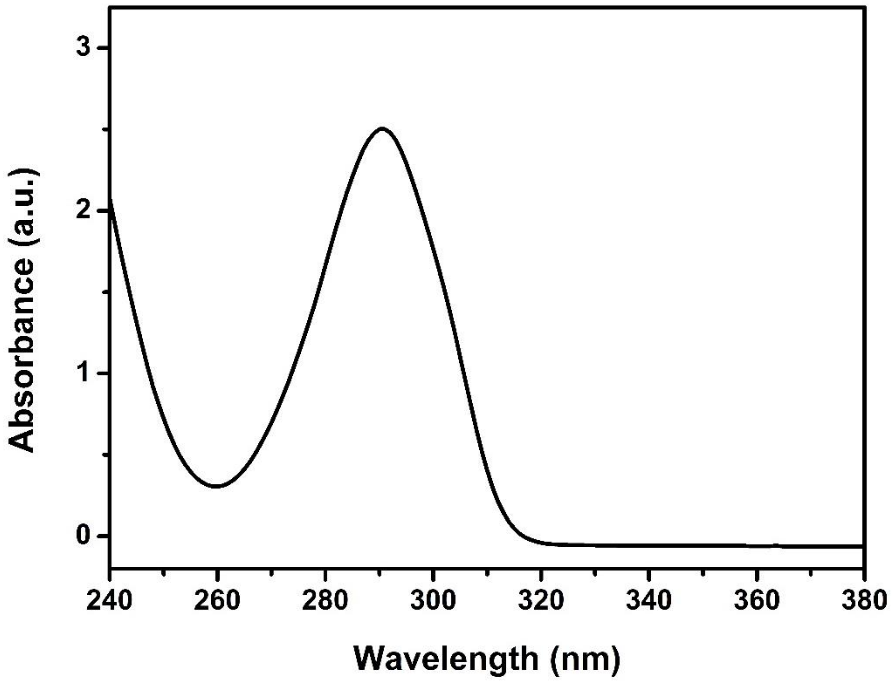

Figure S27. UV-Visible spectra of TEP5.

3.6 ^{1}H NMR spectra H in the presence of increasing concentration of Fe^{3+}

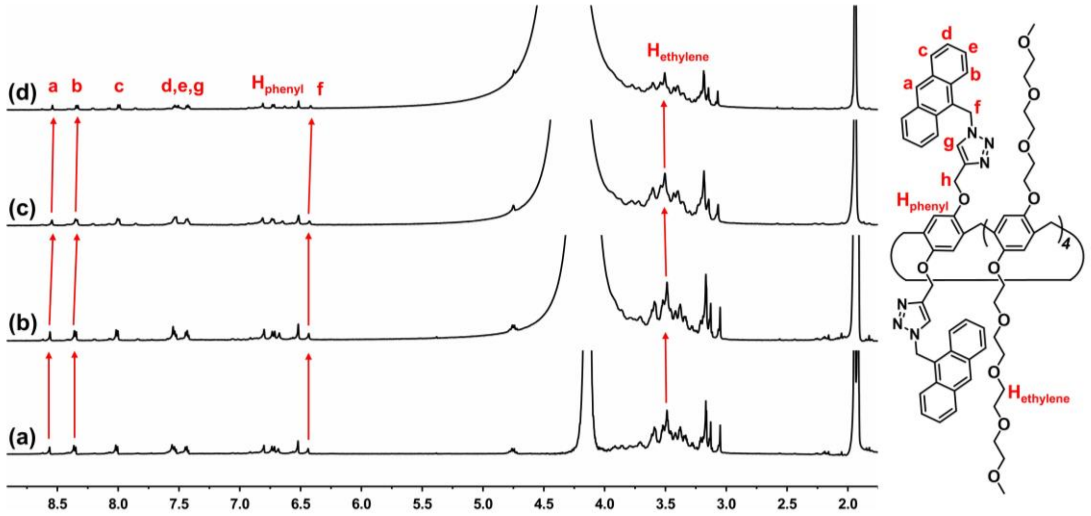

Figure S28. ^{1}H NMR spectra obtained during the titration of **H** with different mole ratios of Fe^{3+} in D_2O/CH_3CN (5:1): (a) 0, (b) 0.5 (c) 1.5 (d) 3.0. The asterisk denotes the solvents.

Table S1. Chemical shift of critical peaks of **H** in the absence and presence of Fe^{3+}

$[Fe^{3+}/H]$	H_a	H_b	H_f	$H_{ethylene}$
0	8.564	8.364	6.438	3.487
0.5	8.561	8.360	6.433	3.487
1.5	8.545	8.350	6.426	3.505
3.0	8.540	8.345	6.418	3.506

3.7 ^{1}H NMR spectrum of TEP5 with addition of Fe^{3+}

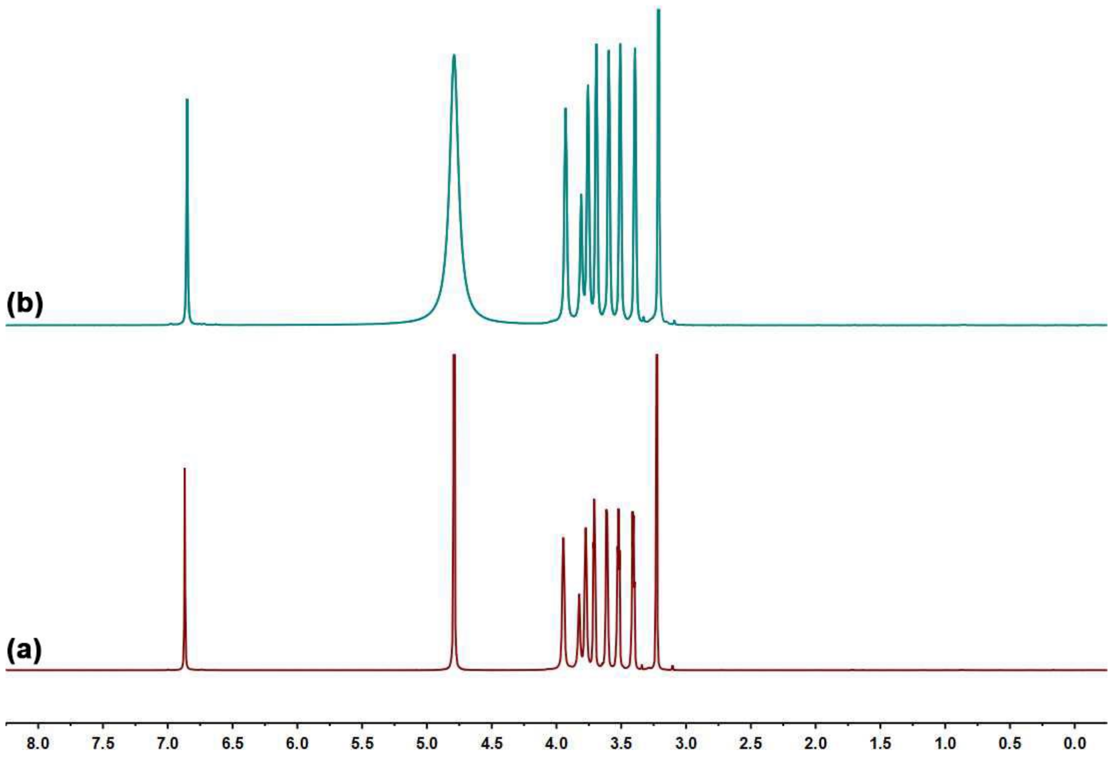

Figure S29. ^{1}H NMR spectra (600 MHz, D_2O) of (a) TEP5 (5.0 mM) (b) TEP5 (5.0 mM) with addition of iron (III) cations (5.0 mM).

3.8 UV-visible spectroscopy of H upon addition of Fe^{3+}

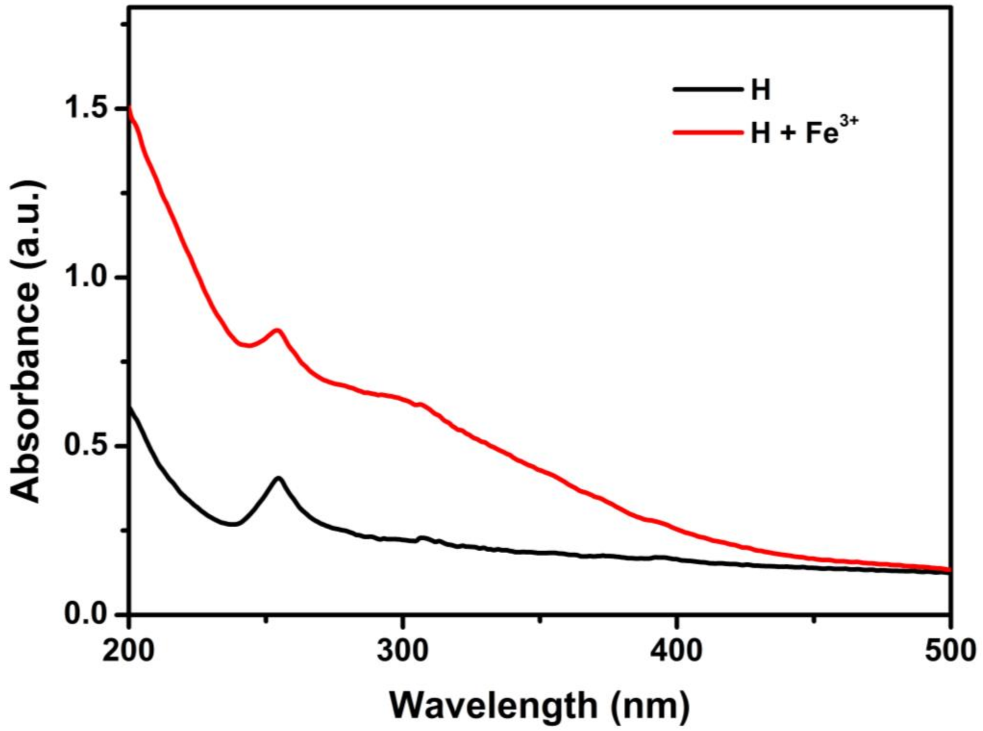

Figure S30. UV-Visible spectra of **H** (20 μM) before and after addition of Fe^{3+}(200 μM).

3.9 Job's plot analysis for H with Fe^{3+}

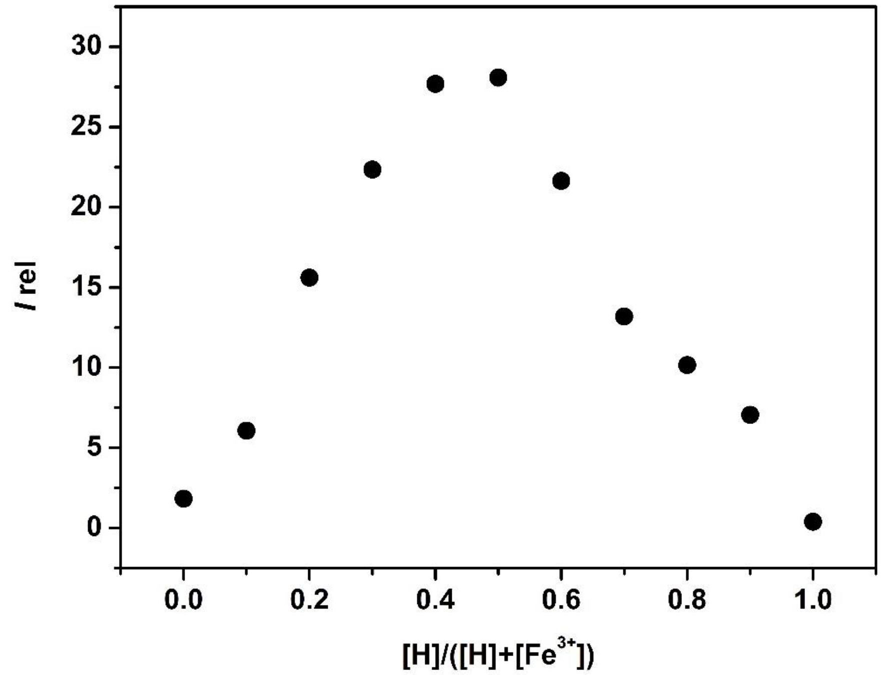

Figure S31. Job's plot for **H** with Fe^{3+} (λ_{ex} = 365 nm, λ_{em} = 413 nm, [**H**] + [Fe^{3+}] = 20 μM).

3.10 Optimized geometries of Fe^{3+} with H

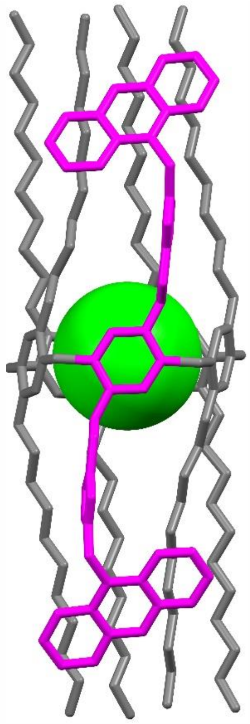

Figure S32. Optimized structure of Fe^{3+}/**H** complex at the B3LYP/6-31G(d) level of theory.

3.11 Fluorescence spectra of H with Fe^{3+} in water

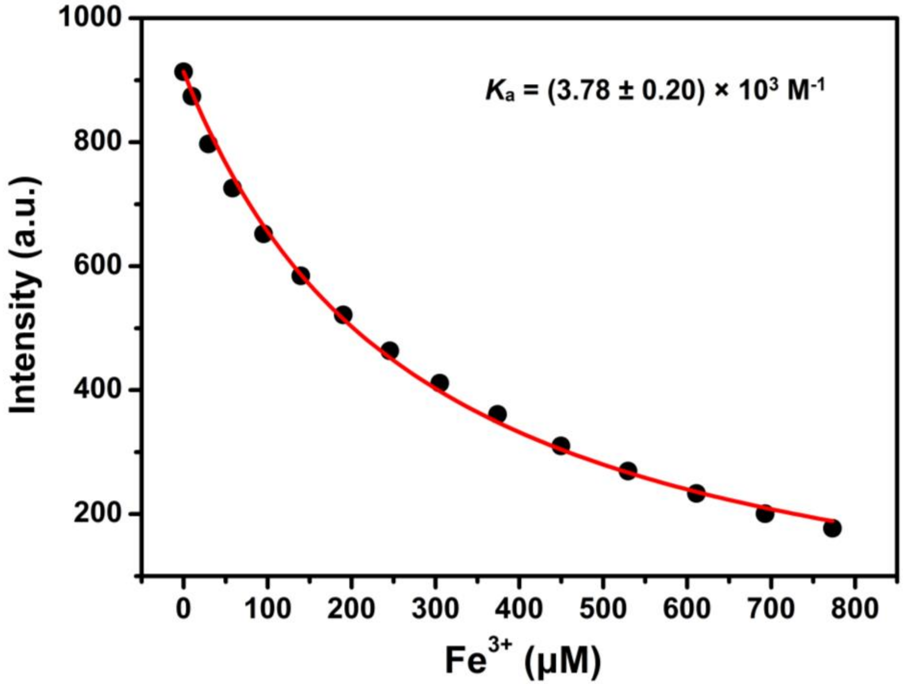

Figure S33. Fluorescence intensity of **H** (20 μM) in the presence of various concentration of Fe^{3+} in water (λ_{ex} = 365 nm).

3.12 Calibration curves of Fe^{3+}

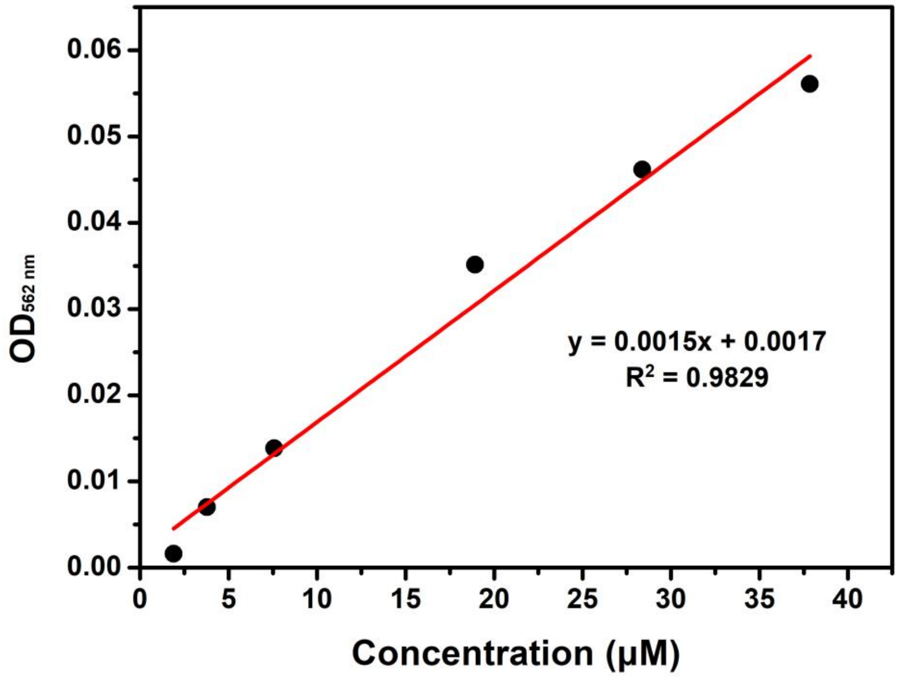

Figure S34. Calibration curve obtained via determining OD absorption and used for calculating the amount of Fe^{3+} concentration.

3.13 Practical application of H for accurate detection of iron concentration

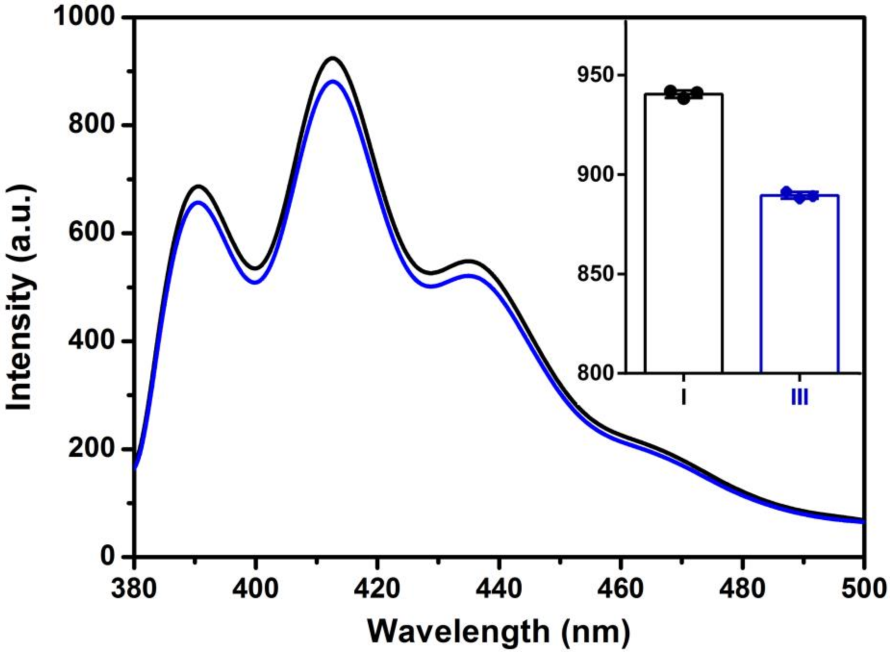

Figure S35. Fluorescence emission spectra (λ_{ex} = 365 nm) adding various ions and Fe^{3+} (5 μM) into **H** (20 μM) in water. Insert: Fluorescence intensity of I (**H**) and III (the mixture of **H** with various ions) at λ_{em} = 413 nm.
